# A participatory systematic review on human rights and the birth of a child with albinism in sub-Saharan Africa

**DOI:** 10.1177/17455057251395420

**Published:** 2025-12-11

**Authors:** Sheryl Reimer-Kirkham, Kendra Rieger, Barbara Astle, Meghann Buyco, Kwame Andrews Daklo, Duncan Dixon, Ikponwosa Ero, Bonny Ibhawoh, Ingrid Tshegofatso Keitseomore, Jennifer Kromberg, Michael Lang, Ronell Leech, Nomasonto Mazibuko, Tumisho Mokwele, Tintswalo Victoria Nesengani, Lillian Ohene, Perpetua Senkoro, Eunice Siaity-Pallangyo, Landa Terblanche, Wisdom Tettey, Mpho Tjope, Lorraine Tshuma, Ingrid Watts, Jessica Wilson, Ramadimetja Shirley Mooa

**Affiliations:** 1 Trinity Western University, Langley, Canada; 2 Africa Albinism Network, Accra, Ghana; 3 Africa Albinism Network, Surrey, Canada; 4 McMaster University, Hamilton, Canada; 5 Reamogeleng Community Organization, Pampierstad, South Africa; 6 University of the Witwatersrand, Johannesburg, South Africa; 7 University of Calgary, Calgary, Canada; 8 University of Pretoria, Pretoria, South Africa; 9 Albinism Society of South Africa, Johannesburg, South Africa; 10 University of Ghana, Accra, Ghana; 11 Tanzania Human Rights Defenders Coalition, Dar es Salaam, Tanzania; 12 Aga Khan University, Dar es Salaam, Tanzania; 13 Carleton University, Ottawa, Canada; 14 Albinism Advocacy for Access, Delportshoop, South Africa; 15 We Are People Foundation, Johannesburg, South Africa

**Keywords:** systematic review, participatory research, human rights, perinatal, albinism, discrimination

## Abstract

**Background:**

The period surrounding birth is a crucial and determining time for many women, particularly for those who give birth to a child with albinism (CWA) due to the stigma, discrimination, and threat to safety they immediately encounter, altering their life trajectory.

**Objectives:**

To synthesize existing evidence on the human rights surrounding the birth of a baby with albinism in sub-Saharan Africa.

**Design:**

We conducted an integrative review through a critical participatory approach. Our review question was; What are the experiences surrounding the birth of a CWA for the mother and father and their carers in sub-Saharan Africa?

**Data sources and methods:**

Our study included 35 academic and 47 gray literature articles and reports (for a total of 82 sources) from 9 academic databases and hand searches with relevant sources. We employed a convergent integrated approach to data synthesis and thematic analysis methods.

Our study included 82 academic and gray literature articles and reports from 9 academic databases and hand searches with relevant sources.

**Results:**

Drawing on African-based perspectives, together with strengths-based, trauma- and violence-informed care, we analyzed the complex lived experiences of mothers who have given birth to a CWA and explored potential sites for transformative change. We identified four themes: (1) *Immediate Experiences: The Life-Defining Moment of Birth* synthesized the experiences and responses of mothers, families, communities, and health providers to a birth to a baby with albinism; (2) *Violent Response to the Birth of a Baby with Albinism* depicted the obstetrical violence, symbolic violence of stigma, discrimination, and social exclusion, gendered and sexualized violence, and violence against the baby with albinism; (3) *Mediating Sites of Structural Violence and Protective Factors* revealed the multiple and interlocking structural sites that deepen the violence shaping the birth experience; and (4) *State as Duty Bearer: Human Rights Obligations and the Policy Determinants of Health* spotlighted the gaps of and recommendations to the States as duty bearers.

**Conclusion:**

Our review revealed not only a matrix of structural violence that characterizes the experience of mothers but also protective factors that become visible with a strengths-based framing.

**Registration:**

Open Science Framework (OSF) registration, DOI https://doi.org/10.17605/OSF.IO/83KMC

## Introduction

Africa, a continent rich in people, resources, and lifeways, is the primary setting of recent attention to the human rights of persons with albinism (PWA).^1^ Women impacted by albinism, particularly in sub-Saharan Africa carry a disproportionate burden of these human rights violations,^2^ and have been described as the hub or “bull’s eye” of the social transformation necessary for human rights to be realized for PWA.^3^ The period surrounding birth is pivotal in setting a direction for safety, security, and well-being. This participatory systematic review aims to synthesize existing evidence (academic and gray literatures) on human rights issues surrounding the birth of a child with albinism (CWA) in Africa. Drawing on African-based perspectives,^4^ such as *Ubuntu*, together with strengths-based, trauma- and violence-informed care,^5^ our intent is to deepen analyses of the lived experiences of mothers who have given birth to a CWA and potential sites for transformative change.

## Background and literature review

### About birthing and disabilities

Quality maternal health is central to the Sustainable Development Goals’ (SDGs) efforts to reduce maternal mortality, particularly in Africa where maternal mortality remains high.^6^ To improve maternal health outcomes, low-resource countries have adopted many of the programs and policies of high-resource countries.^7,8^ Scholars are increasingly advocating for integration of African Indigenous birthing knowledges, including that of traditional birth attendants, as an approach to improve maternal outcomes; reduce high reports of disrespectful maternity care, birth trauma, and obstetrical violence; and foster reproductive justice, birthing sovereignty, and human rights.^7^ Birth trauma and obstetrical violence overlap with disability.^9–11^ Rotenberg et al.^12^ noted that despite relatively high rates of disabilities in Africa, little is known about equity in access to healthcare for women with disabilities who face intersecting challenges such as underemployment and lower education. This gap extends to women living with albinism or giving birth to a CWA.

### About strengths-based and trauma- and violence-informed approaches

Our intent is to avoid deficiency discourses, in line with Tamale’s^13^ call for “the universal narrative of ‘deficiency’ about Africa to be jettisoned for one of dynamism and potential” (p. 1). Strengths-based approaches are gaining ground to support equity and have been explored and defined from Indigenous perspectives.^14–16^ Trauma- and violence-informed approaches uncover “upstream” structures that shape everyday lives of mothers impacted by albinism, and identify protective factors against adverse health and social outcomes that have structural roots. Structural violence refers to how societies are structured in ways that they do harm. Varcoe and Wathen^17^ highlighted the relationship of structural violence to injustice and avoidability, meaning that alternative social arrangements could mitigate (avoid) unfairness and harm, and that all are implicated in the task of rectifying harm. As well illustrated in our analysis, structural violence can operate at interpersonal and systemic levels.

### About albinism, gender, and human rights

Oculocutaneous albinism is a rare, non-contagious inherited group of disorders associated with reduced melanin biosynthesis,^18^ resulting in hypopigmentation (paleness) of skin, hair, and eyes. Many PWA have reduced visual acuity and nystagmus as well as a high risk of skin cancer due to being hypersensitive to ultraviolet rays. Albinism affects people worldwide, with a higher prevalence in Africa. For example, in Tanzania, approximately 1 out of 2673 of the population have albinism, compared to 1 in 17,000 worldwide.^19^

While albinism poses serious health challenges, the most profound impacts often stem from societal reactions and stigma. Misconceptions include that PWA are: contagious; a curse and bring a bad omen to the family and community; a ghost that does not die and thus not human; and magical when their body parts are used in potions or charms.^20,21^ PWA experience human rights violations ranging from bullying, rejection, and discrimination to immediate threats to their life.^22^ In 2015, the United Nations Council responded by approving a mandate on albinism, appointing the first Independent Expert on the Enjoyment of Human Rights by PWA, Ikponwosa Ero.^23^ Our research-advocacy-policy network (see: www.motheringandalbinism.com). supports the UN mandate through collaborative research initiatives in South Africa, Tanzania, and Ghana. Our foundational research has (i) revealed intersecting patterns of human rights violations against mothers and their children starting at birth, (ii) the resilience and determination of mothers as human rights defenders, (iii) how peer support and income-generating groups are avenues to a basic level of human rights, and (iv) how communities such as faith institutions can facilitate the promotion of human rights. Our 4-year project (Canadian Institutes of Health Research Project Grant, 2022-2026, #481405) explores how perinatal experiences of mothers who give birth to a CWA can be improved through health services, health professions education, and the development of equity-oriented and contextually relevant educational strategies, with the overarching aim of promoting, protecting, and fulfilling their human rights.

## Methods

Using a critical, participatory approach, we conducted a systematic integrative review of existing literature,^24,25^ which accommodated inclusion of theoretical and empirical literature using varied methodologies.^26^ Our review question was: *What are the experiences surrounding the birth of a CWA for the mother and father and their carers (families, care providers/birth attendants) in sub-Saharan Africa?*

**Figure 1.**
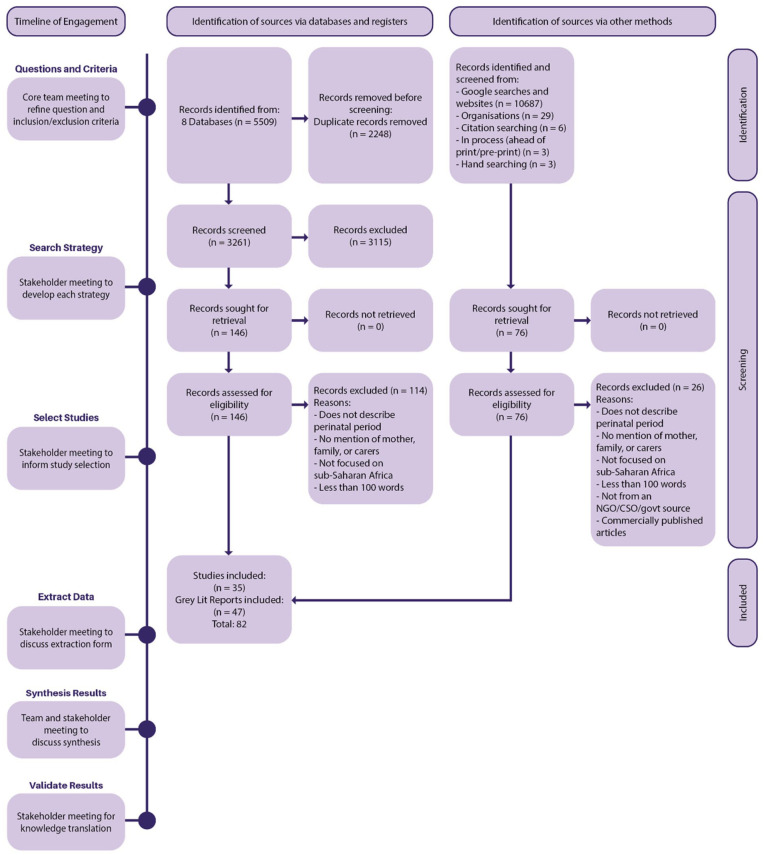
Timeline of engagement and flow diagram (Preferred Reporting Items for Systematic Reviews and Meta-analyses – PRISMA).

As integrative reviews can include interest-holder consultations, we collaborated with PWA, mothers of children with albinism, healthcare workers, and advocates. These consultations fostered relational approaches that blended distinct perspectives, enhanced the meaningfulness and usefulness of our findings^27^ and prevented the dominance of Western ways of knowing. We held six online engagement meetings, and one in-person gathering, with African and Western academics and interest-holders throughout the planning and conduct of the review (see Figure 1). We developed a shared understanding of our intentions and focus, refined our inclusion/exclusion criteria and search strategies, determined information to be gathered, and validated our analysis and findings.

### Search strategy, study selection, quality appraisals, and analysis

A healthcare librarian (DD) developed our search strategy in consultation with research team members and interest-holders (see Supplemental File: Search Strings). It involved four key concepts: (1) sub-Saharan Africa, (2) albinism, (3) birth attendants, and (4) perinatal. Academic and gray literature sources were included which met our criteria of:

1. Addressed the phenomenon of a child born with albinism during the perinatal period in any birthing setting.
2. Described the experiences/perspectives of mothers, fathers, family/community members, or birth attendants such as midwives, nurses, doctors, and traditional birth attendants who were present during the birth of a CWA.
3. Located in the sub-Saharan Africa context.
4. Utilized various types of study designs such as qualitative, quantitative, and mixed method studies, as well as evidence syntheses and discussion-based and theoretical literature.
5. Gray literature: Published by a reputable organization such as government, non-governmental organization (NGO), non-profit organization, civil society activist, or civil society organization (CSO).
6. Limited to English and French due to the feasibility issues with translating articles into English.
7. Hundred directly relevant words or more to have a sufficiently substantive description of mothers and carers experiences of a birth of a baby with albinism.

Sources were excluded if discussion of birth experiences was only in the background section or not a focus of the article. Commercially published articles (i.e., magazines and newspaper articles) and social media posts were also excluded. No time limits were applied, given the relative recent attention to albinism and human rights. We ran our searches of nine databases between the dates of March 10, 2023 and June 28, 2023. No date limit was applied. Languages included English and French. Key websites were searched for gray literature and online journals were hand searched (see Supplemental File: Search Strings and Protocol).^26^ Forward and backward searches with included articles identified additional relevant articles. Retrieved articles were imported and deduped in Covidence^TM^.^28^ Two independent (MB and IW) reviewers screened titles/abstracts of retrieved articles against the inclusion criteria and read the full text of potentially relevant articles to confirm eligibility. Conflicts were resolved by one of the principal investigators.

Two reviewers (MB and IW) conducted quality appraisals using the appropriate Joanna Briggs Institute (JBI) Critical Appraisal Tools (Qualitative, Systematic and Text and Opinion appraisal forms).^29–32^ For gray literature, we used the Public Health Ontario’s^33^ Guide to Appraising Gray Literature and the Authority, Accuracy, Coverage, Objectivity, Data, and Significance Checklist.^34^ Articles were not excluded based on quality, but quality appraisals were considered during analysis (see Supplemental File: Quality Appraisals). The strength of the overall evidence was evaluated using JBI’s Level of Evidence for Meaningfulness.^35^

One reviewer extracted standard categories from included articles and placed data into a Matrix (see Supplemental File: Included Articles), which was checked by another reviewer.^36^ Included articles were imported into NVivo™^37^ for analytic data extraction specific to our review question (see Supplemental File: Data Extraction Forms and Supplemental File: Codebook). The review team employed content and thematic analysis methods,^24,25^ which is consistent with integrative review methods, to identify, analyze, and report patterns within the data. An iterative analysis process began with an inductive approach and evolved to reflect *Ubuntu* and trauma-and violence-informed framings.

## Findings: Description of sample

The academic database search retrieved 5509 articles, and after screening, we included 35 academic articles and 47 reports (total *n* = 82) from the databases and gray literature searches (see Figure 1 for selection details, as well as supplemental materials). After 2248 duplicates were removed, 3261 titles/abstracts were screened against inclusion criteria, and 3115 articles were excluded. The remaining 146 articles were read in full, and 114 were excluded (see Figure 1 for reasons). The academic articles comprised qualitative (*n* = 18) and synthesis (*n* = 1) designs, and discussion articles (*n* = 16). For gray literature sources, 25 were reports produced by NGOs (e.g., UN), and the rest were thesis and dissertations (*n* = 8), book chapters (*n* = 7), and essays and educational materials (*n* = 7). Of the included sources, 69% were published in the last 10 years. Authors represented fields associated with health, social sciences, education, philosophy, religion, and law. The majority of the academic literature had first authors from the Global North (*n* = 23, 66%). Of the 82 sources, only 6 articles had a substantial focus on the experiences at birth and the first year of the child(ren)’s life.^38–44^ The remaining sources contributed to specific aspects of analysis with interpretive and contextual insights (i.e., as latent data).

## Findings: Synthesized interpretive themes

Our analysis provides detailed, synthesized evidence of the *immediate experiences* of giving birth to a CWA (Theme 1), *multi-site violence* in response to the birth of a CWA (Theme 2), and the mediating *structural factors* that shape these experiences (Theme 3). Through a human-rights lens, we probed the included literature not just for individual-level responses, but more so for *state responses*, given their position as duty bearers (Theme 4). Our human rights approach is based on applicable international, regional, and national human rights principles and norms. When the data are read through strengths-based and trauma- and violence-informed approaches, a matrix of structural violence becomes apparent across the themes as a mixing of *obstetrical violence*, *gender-based and infant violence*, *symbolic violence*, *and the institutional violence of inadequate health and education systems*. Symbolic violence, a concept built on Pierre Bourdieu’s symbolic power, refers to non-physical forms of violence that operate through the distortion of symbols, ideas, and beliefs. It is a social form of power that circulates at the level of culture, shaping individuals’ beliefs and behaviors. Symbolic violence is particularly effective because it is often internalized, leading individuals to accept and reproduce social inequities. It is also internalized by those who experience it—such that individuals (PWA in our analysis) come to see the existing social order as natural and inevitable.^45,46^ These intersecting acts of violence are inextricably embedded in historical structural issues, colonial legacies, and neocolonial realities of Africa^47^ that are offset and resisted by Africa-based epistemologies which open space for strengths-based interpretations to emerge (see Table 1 for structure of thematic analysis).

**Table 1.** Interpretive themes and sub-themes.

Themes	Sub-themes
Theme 1: Immediate experiences: the life-defining moment of birth	• “*What’s this lady producing?*”: Experiences and responses of mothers and their birth attendants • “*Bibi had albinism too*”: Experiences and responses of fathers, families, and communities
Theme 2: Violence as response to the birth of a child with albinism and moments of resilience	• “It all starts in the delivery room”: Obstetrical violence • “*Albinism sticks on the woman*”: Gendered and sexualized violence • “*What shall we do with the baby?*”: Infant violence and infanticide
Theme 3: Mediating sites of structural violence and potential transformation	• “*Localized beliefs and postcolonial policies*”: Neocolonialism underpins structural violence • “*Ideologies that sustain*”: Explanatory frameworks perpetuate symbolic violence • “*Assemblages of care*”: Health systems deepen institutional violence or serve as protective factor • “*Include albinism in the curriculum*”: Health professions education maintains institutional violence or acts as protective factor
Theme 4: State as duty bearer: human rights obligations and policy-mediated determinants of health	• “*It’s a pity the government doesn’t help us*”: State obligations and the spectrum of government involvement • “*Not for lack of legal frameworks*”: Human rights and the birth of a child with albinism

### Theme 1: Immediate experiences: the life-defining moment of birth

There was remarkable consistency as to the nature of experiences surrounding the birth of a CWA, particularly for mothers. For them, the life-defining moment of birth activated a trajectory of precarity (we draw on Judith Butler and Simon During to define precarity as “the insecurity of all those who live without reliable and adequate income or without papers, as well as those with no, or unstable, access to the institutions and communities best able to provide legitimacy, recognition and solidarity’ (p. 58)^48,49^ or a trajectory of flourishing. Negative responses of fear, stigma, blaming, and abandonment set the mother on an all-too-common trajectory: tenuous attachment,^38,50^ deepened precarity,^42^ the constant threat of violence,^41^ and an overall experience of a traumatic birth.^40^ In contrast, respectful approaches and explanations about the genetic nature of albinism set a mother on a positive trajectory. Less than half (46%) of included sources reported positive experiences, and when they did so, the positive story was the minority storyline.

#### “What’s this lady producing?” Experiences and responses of mothers and their birth attendants

Spiritualized misunderstandings, gendered violence, and structural vulnerabilities too often marked the timeframe immediately following delivery.^42^

##### Mothers’ initial responses of shock and dismay

Unless a mother had previously given birth to a CWA, their responses were that of shock and dismay instead of the anticipated joy at the arrival of a healthy child.^40–43,51–60^ Of 134 mothers’ first-person accounts in the 6 primary studies,^38,40–44^ there was unanimity in this initial response. Authors described mothers struggling with or being disturbed by the appearance of a visibly distinct child,^38,41,51,54,58^ as they knew “*no other child like that in the entire village*” (p. 7).^54^ Some mothers had never seen a CWA, and they reported feeling afraid and not knowing what “*kind of thing*” they had given birth to (p. 3).^43^

One article provided insight into the immediate and pervasive worry of mothers about the anticipated responses of their families and communities, which amplified the initial shock and dismay. Even before mothers left the hospital or birthing center or place of birth, they anticipated the social and gendered stigma they would encounter.^41^ Disbelief and denial could follow, and led to avoiding caring for the child^41,61^ who did not appear to be their own (e.g., avoiding breastfeeding and eye contact),^20^ and delayed attachment.^43,50,62^ Some authors described how mothers withdrew from community life^2,43^ or kept the child hidden.^42,52,53,63^ Actions such as adopting the child out^38^ or abandoning the CWA^43,62,64,65^ were noted, though uncommon. Over time and if supported, however, mothers usually came to accept and bond with the child.^38,41–43,60^ The literature does not address the long-term mental health impacts of the trauma event of giving birth to a CWA.

##### Birth attendants’ neglectful care and value of respectful support

Crucial to acceptance and a subsequent parenting trajectory was the immediate response of the birth attendant. A third (27 of 82) of the sources mentioned birth attendants’ responses and of these, 96% (26 of 27) reported responses that reinforced parents’ fear and shock or reported disrespectful care.^2,39–44,51,52,54,58–61,64,66–75^ Authors described birth attendants’ fear as extending to the mother herself,^2,40,42,43^ such as when: “*The nurse saw the CWA coming out and shouted, ‘What’s this lady producing?*’” (p. 7).^54^ Gendered blame, such as accusation of infidelity and sleeping with a White man, was routinely directed toward the mother by birth attendants,^21,23,39,58,60^ even at the moment of delivery.^2,42,53,64,76^ An author wrote, “*Mothers described hearing nurses whisper to each other, display expressions of shock and fear, literally run away and scream*” (p. 141).^42^ Underlying the inadequate provision of health information was birth attendants’ own lack of knowledge of how to respond positively when a child is born with albinism.^68^ Without such knowledge, even highly educated professionals reacted with fear, discrimination, and negative comments about the condition.^26,42,69,70^ In Daklo’s^61^ words: “*The nurses’ reactions paint a picture of societal prejudices and misconceptions about the condition fuelled by lack of information*” (p. 58).

Along with reporting birth attendants’ reactions to the birth of a CWA—whether supportive or stigmatizing—as setting a trajectory for the future, providing accurate health information in the early postpartum period was vital. Mothers wanted to know more about albinism and what causes it to educate the community on how to keep their child safe.^39,63^ Eighteen sources (22%) reported patients receiving no education, rudimentary information about albinism, or incorrect information.^54–56,72^ For example, sometimes birth attendants provided “*God only knows*” *as* the cause of albinism. Birth attendants’ vagueness about the child’s condition resulted in mothers’ distrust and confusion,^40,41,44,69^ decreased bonding,^38^ and increased maternal and family anxiety and depression.^38,76,77^ With dire consequences, mothers and their children with albinism face increased social estrangement and violence as they are not equipped to confront stigma, misinformation, and harmful beliefs^77^; and children may experience early skin damage if their parents do not know how to provide for sun protection^41^ (see Supplemental File: Albinism Education).

#### “Bibi had albinism too”: Experiences and responses of fathers, families, and communities

The included sources consistently identified the (predominantly detrimental) impact of the responses of fathers, families, and communities on the safety and security of a mother and her CWA. One author observed “*Emotions of hate that stick on albinism . . . can instigate misogyny, which always sticks on women*” (p. 40).^42^ Yet, these same kin networks could create a powerfully accepting community for the arrival of a CWA, especially when a history of albinism could be traced through family lineage.^78^

### Responses of fathers

Fathers’ reactions of surprise and shock were reported to be like those of mothers and birth attendants.^39,50,57,58,66^ Authors discussed instances of fathers who were saddened,^2,38,50,55,79^ annoyed,^51^ and depressed.^80^ Fathers’ reactions demonstrated a lack of understanding about albinism and perplexity about what had happened.^2,42,81^ Lund cited one father as saying, “*After getting that child, I got shocked because there was no other child like that in the entire village . . . community members always said I gave birth to lubaale [a ghost]*” (p. 3).^66^

Many authors wrote that a common reaction of fathers at the time of birth was accusing the mother of infidelity,^2,39,42,43,50,55,58,64,71,81^ denying paternity,^2,38,42,55,56,70,81^ or denying any contribution to the child having albinism.^40,42,51,54,58,64,82^ The most reported impact was abandonment and/or divorce,^2,55,62,75,83^ placing the burden of care solely on the mother or another close female relative.^68^ Ackley^71^ reported that 90% of children with albinism are raised by single mothers. Social pressure for the father to leave the mother or child was a reality for many families and came from the family or the community.^2,42,52,65,77,84^ Strobell^42^ stated that the fathers’ “*ability to immediately deny paternity when the baby’s skin color is so opposite their own makes this abandonment altogether too easy within the context of existing myths and beliefs*” (p. 142).

Some fathers chose to stay with the mother but demonstrated ambiguous commitment such as being unsupportive despite cohabitating, withholding financial support,^39,42,65,75^ or barely acknowledging their CWA.^42^ Fathers who accepted the child resisted pressure to leave^43,84^ and decided to stay based on love or responsibility.^54^ Fathers were reported as having to process various emotions to reach the stage of accepting the child, which might occur around the same time as the mother does, or later.^85^ Having the support of the father, especially at the time of birth, was a crucial factor in a positive trajectory and required confidence and courage on the part of the father. Bradbury-Jones^54^ quoted one father as saying:

Some people advised me to chase [leave] the wife but I kept on refusing since I love my wife. My wife later gave birth to the second child without albinism, so I continued being strong. After a year she gave birth to twins and both with albinism, so people again stressed I should chase my wife. I asked myself where my wife would go with all those children, so I decided to just keep strong (p. 9).

Only two of the six primary articles^38,43^ incorporated fathers’ perspectives in first person. Research is needed to gather fathers’ firsthand experiences and to develop strategies for their involvement and support.

### Responses of family and community

The pattern of initial shock and dismay at the birth of a CWA carried through to the reactions of family and community—such a birth was perceived as traumatizing to the entire family.^2,42,44,50,70,72^ Community and family members too engaged in gendered blaming with accusations of the mothers’ infidelity^54,58,77^ and of her causing albinism in the child,^43,64,86^ which defiled their lineage.^42,52,55,82,87^ An extended family might banish a mother and child, sending them back to their maternal community.^39,42^ Social exclusion resulted in a devastating lack of social support and economic hardship for mothers and their children. In the context of African *Ubuntu* philosophy which espouses “*I am, because we are*” (p. 3),^54^ a collective social suffering results and is represented by the responses to a birth of a CWA, not on account of the condition of albinism, but on account of widespread misinformation and violence. Not just the mother and child suffer, although they bear the brunt, entire communities and societies suffer.

While reports of negative family/community responses far outweigh those of positive responses, the literature provides a clear picture of how their support can be transformative.^39–41,52,58,82,88^ Acceptance from even one relative instilled general acceptance within the family system.^42,58,78^ As one participant stated, “*in the end, all of them accepted me when they discovered that the sister of my grandfather on our female family side (bibi) had albinism too*” (p. 235).^78^ Having family members with albinism helped with sense-making and acceptance.^42,52,57,58,76,78,86^ The significant role of grandparents in the acceptance^62,70,77^ or rejection^81^ of people affected by albinism was highlighted. In one situation, “*A young man stated that his mother wanted to kill him, but his grandmother protected him*” (p. 43).^70^

Some authors referenced additional community support from faith communities^41,57,60,89,90^ and peer support.^2,42,58^ As example, one congregation was transformed following an “*Understanding Albinism*” seminar and church members started spending time with PWA.^91^ Other faith communities viewed albinism as a curse,^89^ were less supportive, and did not speak up against mistreatment and violence.^57^ As noted by one pastor, “*I honestly have not paid much attention to the issue of the church and albinism. The church is too silent. . .*”^57^ Peer support groups, often under the auspice of a local NGO, provided support groups, education, and capacity building, which in turn mitigated stigma and its effects.^2,41,42,58^ As one worker shared, NGOs combatted the “*the isolation, loneliness, and depravity that so often accompanies the albinism story*” (p. 121).^42^ One mother shared that “*these organizations provide valuable information and give hope because in most cases, they share a lot of positive thoughts, organize events, and share success stories of PWA*” (p. 99).^58^

### Theme 2: Violence as response to the birth of a CWA and moments of resilience

Explicit in the evidence about responses to the birth of a CWA was the presence of violence: as obstetrical violence; as the symbolic violence of stigma, discrimination, and social exclusion (reflected in Theme 1); as the gendered and sexualized violence of blame, abuse, and abandonment; as violence against the child with many references to infanticide. These violence operate at the interpersonal level but must be understood as enmeshed with structural forms of violence (Themes 3 and 4).

#### “It all starts in the delivery room”: Obstetrical violence

Although none of the sources used the language of “obstetrical violence,” when this concept is applied to the data on the immediate experiences of mothers and responses of birth attendants after the birth of a CWA (Theme 1), a framing of obstetrical violence is accurate. O’Brien and Rich^92^ defined obstetrical violence as:

Harm inflicted during or in relation to pregnancy, childbearing, and the post-partum period. Such violence can be both interpersonal and structural, arising from the actions of healthcare providers and also from broader political and economic arrangements that disproportionately harm marginalized populations (p. 2183).

Common acts of obstetrical violence in our data were physical abuse, non-confidential care, non-dignified care, discrimination, and abandonment.^93^ Much of the mistreatment resulted from systemic issues, such as poor education of birth attendants and the lack of resources such as workforce and space that led to rushed and rudimentary care experienced as dehumanizing and disrespectful. O’Brien and Rich^92^ cited as example Kruk et al.’s^94^ report that 19%–28% of women in eight facilities in northeastern Tanzania experienced disrespectful and/or abusive treatment from birth attendants during childbirth. Ojilere and Saleh^95^ concluded that women giving birth to a CWA are significantly more worse off in comparison. Although only two articles directly reference Respectful Maternity Care,^41,60^ several authors emphasize the importance of respect and dignity during childbirth.^41–43,60^

#### “Albinism sticks on the woman”: Gendered and sexualized violence

Virtually all sources mention discrimination, stigma, blame, abuse, and abandonment faced by mothers of a CWA, though not consistently named as gender-based or intimate-partner violence (World Health Organization defines violence against women as “any act of gender-based violence that results in, or is likely to result in, physical, sexual, or mental harm or suffering, including threats of such acts, coercion or arbitrary deprivation of liberty whether occurring in public or in private life”).^96^ Of the 82 sources, only 13 reference or apply a framing of “gender equality” or “gender justice.”^2,21,41,42,58,60,64,70,95,97–100^ Without such framing, the gravity of the situation with its pervasive and deep psycho-trauma^82^ may be overlooked and opportunities for intervention missed. There is evidence of abuse within their marriages or by their partners including psychological and mental torture from insults and isolation^101^ and physical abuse.^21^ Reports also include family members’ abuse of mothers.^21,40,52,53,77^ When reported to police, such incidents are rarely prioritized for investigation.^21^ Offsetting the reality of intimate partner and gender-based violence were inferences to the “strong black woman” motif and her resilience. Fourteen sour-ces^2,41,42,50,54,60,69,73,80,82,90,100,102,103^ use the terminology of “resilience” which itself can implicitly weaken a gender justice lens if the focus shifts too far from systemic forces and instead slides into individualism. The literature includes some reference to Afrocentric womanist theories as helpful in understanding the communitarian nature of resistance to harm (see Strobell’s^42^ application of Hudson-Weem’s^104^ ecological framework; and Ibhawoh et al.’s^90^ discussion of women reclaiming community).

#### “What should we do with the baby?” Infant violence and infanticide

In addition to the gendered abuse experienced by women who give birth to a CWA, infanticide is mentioned in 46% (*n* = 38) of the sources, and more so in the gray literature than the academic (see Table 2 for examples of references to infant violence). To interpret the prevalence of these mentions of infanticide (along with threats, attacks, mutilation), we examined firsthand reports in our primary sample. Only two of the 134 mothers reported direct infant violence: a case in Tanzania of a kidnapped child having a finger cut off^42^; and in a second instance, a birth attendant asked the grandmother what to do with the child:

They did not say anything to me or show me my baby but went straight to my mother and asked what they should do with the baby . . . maybe some people ask midwives to kill their children when they are born with albinism (p. 4).^40^

**Table 2.** References to infant violence and infanticide.

Perpetrators of violence	Examples from the literature
Mothers	• A woman killed her child after the husband’s family threatened to excommunicate her if she did not do so^77^
Birth attendants and health workers	• Choking a CWA so it does not live^57^ • Traditional midwives kill children with albinism and then declare them stillborn and secretly bury them,^71,74,77^ or advise mothers to asphyxiate or starve the child^65^ or as too weak to survive^50^ • Rumors that in some communities, health workers have been involved in exchanging children born with albinism born in labor wards and that hospitals have offered up free sunscreen to attract PWA so that they can be sold or killed^39^
Fathers	• Fathers’ stated desired to kill the infant^55,56,79^ or actually killing the infant^105^ • Fathers encouraged the mother to kill the infant but was not charged^52,62,106^
Community and family members	• Abandon the baby to die^53,58,62,106,107^ • Putting children in a cattle gate to see if they will survive and be allowed to live^84,88,108^ or dropping the infant in a lake to see if it will survive^58,108^ • Community and family members’ attempts to kill the child were reported by several authors^39,62,109,110^
Witchdoctors	• Witchcraft contributes to the killing of PWA as witchdoctors reportedly use body parts of PWA for their rituals^61,71,72^
Criminals and contract killers	• Infanticide by contract killers^20,71,73^

CWA: child with albinism.

Sources referencing infanticide.^1,20,21,40,42,44,52–54,58,65,68,70–75,77,78,80,84,88,89,95,97,105–116^

Even without direct violence, however, a large majority of the mothers expressed a constant sense of fear about the safety of their children. One mother spoke of her deep concern, “*I do not sleep normally as you people do. I am scared especially at night thinking of what might happen when I am asleep*” (p. 38).^39^

A handful of other sources reported firsthand accounts of infant violence (i.e., direct reports^55,82^); the remaining references involve the testimonies of adult PWA who themselves were attacked or threatened as young children^20,109^; news stories of attacks^53,62,71^; hearsay or secondhand accounts^20,53,108^; and official reports.^21,74,111^ Several sources note that infanticide has been practiced by many societies in Africa in the past with some societies continuing the practice today.^52^ The many references to infanticide—although many were hearsay—must be taken seriously, in part because they add to an overall experience of threat and violence.

### Theme 3: Mediating sites of structural violence and potential transformation

The literature is varied in the extent to which birth experiences are interpreted through critical structural lenses. We intensified our analysis with careful attention to root (underlying) causes of individual experiences and responses, and explicated these deeper understandings as for influence and intervention. This interpretive frame of structural violence as avoidable impairment of fundamental human needs is oriented toward identifying sources of structural violence impacting birth experiences, such as historical relationships (e.g., neocolonialism), ideologies and beliefs (e.g., explanatory frameworks and symbolic violence), and social institutions (e.g., healthcare systems and health professions education).

#### “Localized beliefs and postcolonial policies”: Neocolonialism underpins structural violence

Colonialism (both historical imperialisms and contemporary geopolitical exploitations) and coloniality operate in the background, which Tamale^47^ described as the psychological aftermath of colonial domination, racism, and dehumanization on the human psyche. We posed the question “*How does the selected literature on birth experiences acknowledge or denounce the impact of colonial(ity) on the perinatal experience of mothers of a CWA*?” Thirteen (16%) sources briefly cite colonial history as context,^42,54,68,70,71,79,86,88,95,111,115,117,118^ such as referring to colonial suppression of Indigenous knowledges,^54,88^ global relations such as post-colonial structural adjustment policies that have eroded government care^68,71,86^ and drive the “occult discourses and practices” of the trade of the body parts of PWA,^71^ outdated colonial laws on witchcraft,^111,115^ and colonial histories of apartheid based on colour.^70^ One source^71^ explicates how attacks against PWA are “an intensification of an old story that began in pre-colonial Central Africa” (p. 3), and that understanding violence requires looking “at the localized occult beliefs . . . along with postcolonial structural adjustment policies and the transition to capitalism in a formerly socialist society” (p. 23). The analytic lens of structural violence made visible the intersecting impacts of neocolonialism, showing the need for comprehensive analyses of the historical and political mediators of violence.

#### “Ideologies that sustain”: Explanatory frameworks perpetuate symbolic violence

Anticipating evidence about African-based perspectives such as *Ubuntu* principles as protective factors, we posed the analytic question, “*How does the literature on birth experiences acknowledge, integrate, or cite African Indigenous beliefs?*” Five sources^42,54,57,68,91^ (6%) referred to *Ubuntu*, with only one author^91^ providing a contextualized explanation of the complexities that preclude the praxis of *Ubuntu* be extended to PWA. Explicating African cosmology, Imafidon^91^ explained that PWA in Africa are seen as ontologically different (i.e., that PWA are not human), and this alterity becomes the rationalization to exclude PWA so that harmony and equilibrium can be promoted within a community; “in order to protect the normalcy of things, societies manage to establish and promote ideologies and structures that sustain the status quo” (p. 164). There is thus a paradox in how *Ubuntu* principles are applied, such that the collectivism and responsibility for the community are not easily extended to include PWA because of the presumed threat they pose. Misconceptions about personhood must be corrected in order for *Ubuntu* to serve as protective mechanism for PWA.

The dominant representation of the causes of discrimination and violence tended toward pejorative references to “myths” and “superstitions.” The cataloguing of beliefs could be grouped as natural, scientific, or spiritualized explanations (see Table 3 for causation understandings of the birth of a CWA). Looking at beliefs that relate to *natural causes for albinism* (*n* = 16 sources; 20%), numerous beliefs have been cited to explain the birth of a CWA,^89^ many of which are physiological responses stemming from social interactions with a PWA. While more articles mentioned *scientific/genetic explanations* for albinism (*n* = 22; 27%), these explanations are often reported to be held alongside other explanations.^53,54,64^ As to *spiritual explanations for albinism* (*n* = 24; 29%), these were presented as positive beliefs (e.g., as a blessing), or negative beliefs (e.g., as a curse or punishment) about albinism. Negative spiritualized beliefs often evoked fear and caused people to hate or react violently to PWA.^20^ Negative and inaccurate underlying explanations for albinism, often taken-for-granted and unquestioned, operate as forms non-physical or symbolic violence.^45,46^ Misbeliefs were internalized by those who wielded them and the mothers impacted by albinism who experienced them, such that actions on the misbeliefs might seem natural and inevitable. The selected sources include some insight as to how resistance to symbolic violence may come about through a combination of individual insight and structural protective factors.^41^

**Table 3.** Causation understandings of the birth of a child with albinism.

Causation understandings	Examples from the literature
Natural (neutral) causes (*n* = 16)	- Conception during menstruation^109^ - Lack of iron^78^ - Promiscuity^53,62^ - Sleeping with White man^2,42,52,63^ - “Catch” albinism: touching or looking at a PWA,^53,56,80^ infection^72,80^ - Being rude to a PWA^44^ - Snake inside women^53^
Scientific causes (*n* = 22)	- Genetic origin^65,67,75,76,83^ - Family history of albinism (without understanding underlying genetics per se)^61,78,85,86,103^ - PWA a product of inbreeding^64^
Spiritualized causes (*n* = 24)	- God’s plan or will or gift^43,80,119^ - Curse or punishment from God^51,73,109,117^ - Evil spirits or witchcraft^43,52,61,62,72,105,112^ - PWA are ghosts or “an other” as a different and unusual entity, not a human being^91^ - Woman has slept with a malevolent spirit (Shona folklore Zimbabwe)^53^

PWA: persons with albinism.

#### “Assemblages of care”: Health systems deepen institutional violence or serve as protective factor

We analyzed how healthcare systems are portrayed in the literature as structural influences on the experiences surrounding the birth of a CWA. Brocco^86^ referred to “assemblages of care” whereby PWA access formal biomedical systems (typically government-run hospitals or clinics) in interface with informal care (e.g., traditional birth attendant/midwife at a home birth; NGOs providing early health information; and kin-based care). Whether healthcare was provided by formal or informal means, and in urban or rural settings, it could become a site of institutional violence or serve as a protective factor. Our analysis suggests that even in low-resource settings, relatively simple adjustments to care could result in better outcomes.

##### Under resourced formal health systems

Few sources situated access to healthcare in the political-economic landscape of sub-Saharan Africa regarding why formal healthcare systems are under resourced. Brocco^86^ traced the geopolitical factors that have weakened health systems with Tanzania’s example of the post-independence imposition of the World Bank and International Monetary Fund’s structural adjustment programs. Economic reforms requiring trade liberalization and deregulation caused a “steep reduction in governmental expenditures for healthcare, education, and housing programs” (p. 117). The Tanzanian state increasingly relied on external socioeconomic support and NGOs for services, which are often inconsistently distributed. In our dataset, such geopolitical analyses are largely absent in discussions of perinatal care at the birth of a CWA.

Some evidence (more so in the gray literature), however, connects birth experiences of (i.e., lack of respectful care, health information, and skin and vision care) to underlying political economy. Workforce issues (such as under staffing, need for additional training, and poorly equipped health settings) are cited by eight references^2,39,41,42,60,70,77,90^ as impacting the perinatal experience, though not with in-depth analysis. For mothers affected by albinism, stigma was operationalized through health systems by the stigmatizing attitudes and behaviors of birth attendants, a lack of access to timely and quality health services and lack of health-related information about the cause and care of albinism. In some countries, a differential system was described as to whether the birth was in a public or privately funded center/hospital, with more respectful care and access to specialty resources described in private care.^60^

##### Informal networks of care

There is strong evidence about how CSOs fill the gaps for the healthcare that governments do not provide, along with their contributions in victim support, peer support, public education, and advocacy.^73,77^ Less evidence exists about NGO involvement specific to perinatal care for mothers and their children with albinism. Three examples of CSOs operating as protective factors were cited in the literature: (i) an NGO provided professional development education for birth attendants on a maternity ward in Tanzania^42,90^; (ii) NGOs facilitated early peer support in Tanzania and South Africa^41,58^; and (iii) mobile clinics in TZ provided early skin and vision care.^42,64^

Straddling formal and informal care were traditional birth attendants. Thirty-two (39%) articles^2,39–43,50,51,53,57,60,61,64,65,67,70–73,75,77,88,95,107,110,113,114,120,121^ mention traditional birth attendants (also referred to as midwives) as providing care at the time of birth of a CWA. In some cases these are presented positively. For example, one source^43^ quoted a mother as saying “*the midwife comforted me and told me of others who had children with albinism*,” while Mhando^110^ reported that traditional healers and midwives held a village event to advocate that PWA be well treated. More often the literature associated traditional birth attendants with perpetuating inaccurate views about albinism and as perpetrators of infanticide, especially in rural settings.^2,58,71,88^ As an example:

Medical personnel who deliver babies may attempt to privately kill the infant before the mother is aware of the circumstances, claiming the baby was a stillbirth or late miscarriage. While this might occur out of a sense of benevolence, it also demonstrates the general lack of awareness surrounding albinism, ignoring that a baby with the condition can live a full and happy life” (p. 12).^68^

Given the many home births attended by traditional midwives in rural sub-Saharan Africa,^122^ more research is needed to understand their practice and how Indigenous (traditional) knowledges could support mothers who give birth to a CWA.

##### Geography as determinant of access to care

The strongest theme about healthcare relates to access to care in under-resourced, rural settings. Many sources referenced lack of referrals and access to specialized skin and vision care, recommended in the first year of life,^103^ as inconsistent or unavailable in rural areas. Some mothers were referred to genetic counsellors upon the birth of their child^53,58^ in large regional hospitals^53^ but lacking in rural areas.^41,113^ In a South African study, one-third of mothers had genetic counselling, most often those close to urban centers. In the absence of genetic counselling, some parents were referred to a doctor after the mother gave birth to confirm the child had albinism.^58^ Birth attendants like a midwife or nurse might also provide genetic education to mothers.^41,43^ In some cases, fathers were involved and provided with education.^43^

#### “Include albinism in the curriculum”: Health professions education maintains institutional violence or acts as protective factor

While there are many calls for birth attendant education, few sources^63,123^ provided substantive information (we are concurrently conducting a Rapid Review of the educational resources available to teach respectful maternity care (RMC) about the nature of that education and the system in which the education might be delivered. Some mention is made of what birth attendants (nurses, midwives, doctors) should be taught about the perinatal care related to albinism: genetic causation^39,42^; countering misinformation^39^; supporting mental health^63^; health information about sun protection and eye care^44^; and the importance of interpersonal relationships and respect.^58,123^ RMC was recommended by two sources as an approach that might well improve carers’ and mothers’ experiences.^41,60^ An example is given of the positive impact of continuing education about albinism for birth attendants in Tanzania, specifically in rural areas.^42^ Another source advocates that in addition to midwives, all qualified nurses in maternity departments, antenatal wards, and clinics for children under-5 should have continuing education about albinism.^39^ There is preliminary evidence to suggest specialized genetic counselling be supported by widespread primary genetic health information and clinical genetic outreach programs.^42,60,95^ Imafidon^91^ made the observation that even when birth attendants are well-trained about genetics, they may still be caught in the web of cultural ideologies that underlie myths and stigma. One source identifies the need for more specialists:

In many countries, there is a lack of medical specialists who are knowledgeable about albinism-specific health issues; in particular, there is a lack of knowledgeable dermatologists and other skin care professionals, genetic counsellors, and ophthalmologists and eye specialists (p. 83).^77^

Macro structures play a major role in the education of the health professions, but few of the authors spoke of these structures. The same geopolitical forces that constrain government investment in health systems apply to higher education.^86^ Only three sources reference government duty for birth attendant education.^51,86,99^ There is an implicit assumption that NGOs and CSOs could play a significant role in the education of birth attendants by, for example, offering education seminars on albinism.^67,90^

### Theme 4: State as duty bearer: human rights obligations and policy-mediated determinants of health

In this final theme, we probe sources as to how governments as duty bearers respond to what is owed their citizens (specifically, those who give birth to a CWA and the interest-holders involved). Although individual carers (birth attendants, family, and community members) have indispensable roles in their support to the birth of a CWA (with mothers and the child as HR claimants), a human rights lens extends to a systems investigation of state as a principal duty bearer. A strength of the included gray literature is the consistent spotlight put to what governments should do to fulfill their human rights obligations. Thirty-eight sources^21,23,39–42,44,51,53,54,58,60,63–66,69–74,77,80,82–84,90,100,101,106,108,112,114,115,119,121^ included state-level or government recommendations, most from gray literature. Most sources (61 of 82 sources) referred to human rights in some way, albeit with varying comprehensiveness. We interpret this majority as characteristic of the current emphases in albinism, health, psychosocial- and human rights-related scholarship, particularly since the UN’s appointment of the Independent Expert in 2015.

#### “It’s a pity the government doesn’t help us”: State obligations and the spectrum of government involvement

Our analysis captured policy options (also referred to as levers or approaches) for how governments should respond to the challenge of improving perinatal experiences of mothers impacted by albinism. We adapted Roberts et al.’s^124^ framework to show how these recommendations can be understood on a spectrum from direct state provision at one end, to deferring to non-state actors such^75,107,111^ as NGOs at the other end of government involvement. Although public provision is presumably more difficult in low-resource countries, of the policy options named the majority fall to public provision and legislation. Where national health systems lack supports, NGOs filled in the gaps.^41,73,75^ NGOs can provide some services, but as Buyco et al.^69^ argued, ultimately it is the responsibility of provincial and national departments of health to provide services. Table 4 displays the balance and type of policy levers that are recommended.

**Table 4.** Analysis of policy levers to improve perinatal experiences after the birth of a child with albinism.

Policy lever	Definition	Policy recommendations (with exemplar references)
Public provision (organization) *n* = 20 sources	Direct, *state provision* (*administered + funded system*)	• Implement national health Insurance for universal healthcare that is accessible and free of discrimination^66,83,97,114^ • Albinism-specific: Government coverage for skin, eye, genetic, income assistance, and employment support^39,101,118^; ensure availability of cryotherapy and dermatologists^66^ • Endorse and support in-country sunscreen production^66^ • Gather data systematically upon the birth of a CWA (i.e., birth registry)^121^
Finance and taxation *n* = 6 sources	Revenue generation using *taxation levers* and allocation of resources (*funding)*	• Import glasses without tax^77^ • Offer loans, grants, disability allowance, and tax credits to PWA and their families^54,63,66^ • Provide grants to CSOs so they can support new families^82,106^ • Add sunscreen to a minimum healthcare package for PWA^66^ • Waive import duties on sunscreen^66^ • Provide disability grants^63^ • Governments, civil society, and international organizations to fill the affordability gap^121^
Regulation (legislation) *n* = 19 sources	Enforcement levers through *legislation* and regulation. Law enforcement and access to justice	• Develop and implement National Action Plans on albinism^39,114,115,119^ • Monitor and report human rights violations such as violence, human trafficking, infanticide^66,72^ • Legislate albinism as disability^114,118^ • Pass legislation for data collection on prevalence and health outcomes albinism (e.g., census)^58^ • Pass legislation to address violence towards PWA and to be brought to justice^21,74^ • Pass legislation regarding accessibility support (assistive devices)^60^ • Develop and enforce gender equality policies^60^
Guidelines *n* = 6 sources	Motivation and direction in a government policy or *guideline*	• Develop curriculum and continuing education for midwives and other birth attendants^51,77^ • Implement guidelines Respectful Maternity Care^60^ • Develop resources for prenatal genetic education^58,97^ • Train policing to respond to gender-based^60^ • Include albinism in national cancer strategies^60^ • Ensure guidelines and policies are implemented for assistive devices for CWA and trainings for teachers^60^
Community education *n* = 22 sources	Exhortation and *moral suasion* through spreading of information to influence changes in behavior	• Enact intersectoral collaboration of government with CSOs and communities,^82,108^ such as around law enforcement^41^ • Mount public awareness campaigns by government against witchcraft^73,111^; against misconceptions^83^ and discrimination^72,106^; for dignity and rights^72^; for accurate knowledge^39,107,111^ • Appoint PWA to government positions^72,77^ • Support research^77^ • Support International Albinism Awareness Day^77^
Civil society *n* = 7 sources	State defers solution to private, *NGO sectors* (Non-intervention, non-regulatory)—civil society fills many gaps	• Provide public education by CSOs (e.g., about genetics, countering stigma, discrimination and witchcraft-related violence)^2,41,69^ • Discourage or counter witchcraft^21^ • Promote involvement of PWA in public spaces^2^ • Outreach to PWA to provide support^101^ • Facilitate peer support groups^2,82^ • Foster access to or direct provision of health services, such as skin and eye care^39,41,121^ • Educate service providers^77^ • Contribute to research (e.g., on prevalence of albinism)^77^ • Advocate for: albinism recognized as disability^77^ and disability benefits^41^; employment (Lynch)^80^; education^80^; poverty eradication^41^

CWA: child with albinism; PWA: persons with albinism.

The evidence portrays a situation that is less a policy vacuum than it is an implementation gap.^90^ The literature identified policy vacuums related to lack of a national action plan,^114,115,119^ extending disability grants and funding to include,^60,63^ creating national guidelines for RMC,^60^ developing census and HR monitoring databases,^114^ and incorporating albinism in a national cancer strategy.^60,69^ Implementation gaps (and the correlated consequences) were multiple and at various levels—for example, not implementing SDGs,^114^ not enforcing laws about human rights violations or investigating crimes against PWA,^21^ and not providing access to health.^114^ The World Health Organization’s^125^ framework on *Policy Levers to Enhance Health Workforce Performance for Compassionate and Respectful Care* is instructive as a policy analysis framework specific to the intent of this systematic review (see Table 5 for the application of this framework). The strength of this framework is the multi-level analysis, from individuals to organizations to systems, such that respectful care does not rest solely on the virtues and values of a birth attendant; rather the experience of individuals is contingent upon organizational and systems responses.

**Table 5.** WHO framework for compassionate and respectful care, applied to the birth of a CWA.

Levels for policy levers	Examples of policy recommendations from the literature
Individuals in the system (birth attendant, families)	• Educate birth attendants (on albinism with genetic counselling, disability, gender, witchcraft, visual rehabilitation, skin cancer exams, and treatment)^77^ • Educate families (on sun protection, genetic cause)^41^ • Include father in care and education^41^ • Include information about albinism in prenatal teaching^41^ • Foster meaningful engagement in care decisions^41^ • Educate PWA (on their rights to safety and security)^66^ • Facilitate health and social care (referrals to skin, eye + genetic counselling, mental health including assessment for post-partum depression and maternal attachment)^39,58,116^ • Provide trauma- and violence-informed care^41^
Structures/functioning of the organization	• Provide accessible health services (e.g., genetic clinics, skincare and screening, free sun lotion, cancer treatment (curative and cosmetic), mental health services), and health information. Rural access can be addressed through mobile clinics^41,69,77^ • Ensure adequate staffing on labor wards and in dermatology clinics of competent, informed (about albinism) birth attendants^39^ • Recognize disability status^77,118^ • Provide prompt provision of medical and psychological support to victims of violence^121^ • Create structures for early peer support^41^
Architecture and oversight of system (incl. Leg, monitoring)	• Provide national health insurance and/or albinism-specific funding^82^ • Stable funding: where healthcare is outsourced to CSO, governments to provide funding^82^ • Develop legislation to declare albinism a disability (to increase access to services)^118^ • Promote RMC to all sectors^60^ • Establish or tighten legal safeguards: witchcraft, violence, monitoring^73,115^ • Create data-driven monitoring^44,121^

CWA: child with albinism; PWA: persons with albinism; RMC: respectful maternity care.

#### “Not for lack of legal frameworks”: Human rights and the birth of a CWA

A range of human rights instruments are named in the included sources (see Table 6). However, none of these international instruments explicitly relate these rights to birth. Based on our synthesis of the experience of families upon the birth of a CWA in Africa, a pattern exists as to the rights that are most likely to be threatened, particularly the right to be seen as a person with full rights. Though not listed in the included sources, legal authority for this right includes the *International Convention on Civil and Political Rights*, 1966, Article 17, and the Convention *on the Rights of the Child*, 1990, Article 16, 23.

**Table 6.** Threats to rights and related human rights instruments.

Applicable international human rights instruments (named in the included literature)	• Universal Declaration of Human Rights (1948) • Convention on the Rights of Persons with Disabilities (2006) • International Covenant on Economic, Social, and Cultural Rights (1966) • Convention on the Elimination of All Forms of Discrimination Against Women (1979) • Convention on the Rights of the Child (1989) • *African Charter of Human and Peoples Rights (1986)*
Threats to rights during perinatal period	• Right to humanness and life (for the infant, including the right to life and non-violence) • Right to gender equality and freedom from all discrimination • Right to health, including health information and access to RMC • Disability rights in which PWA are recognized as having a disability (enabling them access to disability-related services) • Right to an adequate standard of living (on account of abandonment and precarity • Right to access to justice

RMC: respectful maternity care.

## Discussion

To our knowledge, this is the first systematic review of birthing experiences of mothers with a CWA, and it is further distinguished as a critical participatory review. Its integrative method was helpful in mapping the current state of knowledge (or the field)—what Cronin and George^126^ referred to as sense-making—and offering re-interpretation or sense-giving.

### Mapping current state of knowledge (sense-making)

The life-defining birth of a CWA activates a family’s trajectory of precarity or flourishing. The suffering and trauma faced by many mothers who give birth to a CWA started in the delivery room with their own surprise and fear, which were amplified by stigmatizing responses from birth attendants, a lack of access to necessary health services, and social exclusion exhibited by fathers, family, and community. Too often, structural violence—as a web of obstetrical, symbolic, gender-based and infanticide, and institutional violence—characterized the perinatal period. Under-resourced healthcare systems and health professions education, together with gaps in implementing human rights conventions, contributed to a trajectory of precarity. Though not necessarily drawing on specific human rights principles and norms, many mothers, as human rights claimants and defenders resisted dominant gendered narratives, sought out health information, and engaged in peer support. Service delivery and government responses are needed to address the precarity of mothers and their children with albinism, and must be designed with awareness of, and responsiveness to, the impact of structural violence on their lived realities. In this way, protective factors rooted in Indigenous epistemologies can shield and nurture the family of the newborn with albinism, toward full enjoyment of human rights. Table 7 summarizes key findings, including the nature of the supporting evidence.

**Table 7.** Summary of the state of knowledge.

Conclusions (supported by evidence)	Nature of evidence
1. The perinatal experiences of mothers who give birth to a CWA are rife with violence (evidenced in stigma, discrimination, abandonment, abuse, and precarity)	The majority of included sources report trauma and violence (mostly moderate strength studies—qualitative, discussion, expert opinion)
2. Many birth attendants may not have accurate knowledge about albinism and thus draw on stigmatizing circulating or social knowledges about albinism, rather than providing accurate teaching. This contributes to neglectful practice and obstetrical violence	Some moderate strength studies report uninformed and discriminatory birth attendants. Birth attendant firsthand perspectives have not been explored
3. If mothers receive early (immediate) genetic explanations about the condition and welcome/acceptance to the child as fully human, they are empowered to resist stigma and discrimination. This equipping is crucial as mothers are primary human rights defenders for their CWA	A handful of moderate strength studies give evidence of genetic counselling as best practice. However, access to genetic counselling is limited (primarily to urban South African settings)
4. Fathers may also act upon inaccurate knowledge about albinism, and often respond with blaming and abandonment. They have not been well included in education and research about the birthing experiences of children with albinism	Little evidence exists from fathers’ perspectives. No study was found of fathers’ experience at the birth of a CWA as the primary focus
5. Based on misbeliefs, community members often view the CWA as bringing bad luck and react with fear, discrimination, and social exclusion. Citing family histories of albinism, providing factual information about causation, and leveraging *Ubuntu* principles about respect and inclusion can offset misbeliefs	The majority of included sources report social exclusion (mostly moderate strength studies—qualitative, discussion, expert opinion). Little evidence exists as to targeted interventions to strengthen families and communities as protective factors
6. Violence against babies with albinism continues to be a risk, with continued reporting of attacks and widespread fear	The majority of sources report infanticide and threats against infants. Evidence most often is hearsay rather than empirical, with only a handful of firsthand accounts
7. Health systems are neither sufficiently resourced to provide the access to genetic counselling, mental health support, and eye and vision car, nor to mitigate the structural violence resulting from under resourcing and lack of care. Simple steps such relationships between albinism advocates and hospitals, and employee education about albinism have been shown as protective factors that shift stigma and reduce trauma and violence	Multiple sources report reduced healthcare access (mostly moderate strength studies—qualitative, discussion, expert opinion). Evidence about protective factors through health systems has not been well developed
8. Health professions education is an upstream approach to reducing birth trauma and setting families on a thriving trajectory. Education should foster respectful care for mothers and families impacted by albinism	Little evidence exists about health professions education about albinism
9. Government policies and resources are looked to as protective factors for persons with albinism. Few policies have been developed that are specific to the birth of a CWA	A variety of policy options are recommended but rigorous policy analyses is lacking in the literature
10. Human rights instruments exist to protect the rights of the child born with albinism, and their mother, but these instruments require advocacy, implementation, and monitoring	A robust human rights framework exists related to albinism
Evidentiary gaps	Nature of gap
1. How do traditional birth attendants, traditional knowledges, and *Ubuntu* principles support families upon the birth of a CWA?	Minimal exploration of the firsthand views of traditional birth attendants and their approaches at the birth of a CWA
2. How could RMC improve the experiences surrounding the birth of a CWA? What is the impact of health systems (systems-level) on RMC?	No evidence about how respectful maternity care can reduce birth trauma for mothers who give birth to a CWA
3. What interventions can shift gender based violence (GBV) and intimate partner violence (IPV) that leave mothers precarious?	The data specific to experiences surrounding the birth of a CWA does not provide evidence about interventions to address GBV or IPV
4. What approaches to community engagement and public education about albinism will shift misbeliefs so mothers and their babies with albinism are embraced?	Minimal evidence on innovative strengths-based and family-oriented approaches to community engagement in response to the birth of a CWA
5. How can accessible networks of peer support for new mothers and families of children with albinism incubate community inclusion, emotional support, health education, and advocacy?	Some sources reference peer support groups as helpful, but research is needed to support the scaling up of impactful peer support networks
6. What is the impact of primary-level community-based genetic counselling, such that all health workers (including those in rural areas) are equipped to provide basic genetic information upon the birth of a CWA?	While genetic counselling is advocated, specifically in larger urban centers, there are not models for accessible delivery in remote and low-resource settings
7. What system-wide pedagogies and knowledges can be embedded in health professions education to equip birth attendants for the birth of a CWA?	While the need for enhanced health professions education is supported by research, the “how” and “what” of the education needed requires more study
8. What is the effect of civil society involvement in situations where there are government gaps in services?	Many albinism advocacy groups have been established in the past decade, and research is needed to understand and maximize their contributions
9. What metrics are needed to provide evidence of the impact of interventions targeted to strengthen protective factors for mothers impacted by albinism?	In anticipation of intervention research, measurement tools such as patient-reported experience measures related to the birth experiences of mothers of children with albinism or more broadly, children with other disabilities, are needed
Implementation gaps	Nature of gap
1. Consistent provision of respect and health information (incl. genetic counselling and trauma- and violence-informed care) at the time of birth	Evidence exists as to what should be taught and how mothers/families should be treated. Development and implementation of clinical guidelines and widely available health information (e.g., brochures, online resources) are required
2. Specialized care (ophthalmology, dermatology, genetic counselling) that is accessible, including in remote settings (by innovative means)	Few countries in Africa have sufficient specialists, especially in rural areas. Mobile clinics, remote access, and primary care can scale up implementation
3. Although states are looked to as duty-bearers, geopolitical (neocolonial) influences severely constrain the availability of resources to be put to health and social services and higher education	Albinism-specific strategic priorities should be developed that maximize impact for modest expenditures. For example, the cost of preventative services (free sunscreen that is manufactured in-country) will quickly offset, in the long term the expense of more costly skin cancer treatment
4. State accountabilities to provide for their citizens’ human rights (focused on safety and security)	Legislation for data collection (mechanisms for central birth registries), and monitoring for safety and security. Monitor human rights violations, and prosecute such infractions
Theoretical gaps	Nature of gap
1. Health-related literatures on albinism and human rights tend toward individualism and micro-level analyses	Critical, social analyses are required to reveal underlying causes of systems and structural violence. For example, a political economy lens to analyze in contextualized ways the pathways and mechanisms through which power configurations cause health inequities (see Lynch). Trauma- and violence-informed approaches provide conceptual tools to highlight the relationship of structural violence to injustice
2. Although gender justice is central to the birthing experiences surrounding the birth of a CWA (impacting mothers, fathers, and birth attendants), more common are interpretations that name myths, superstitions, and discrimination without links to gender justice. The influence of patriarchy—even in the encounter between a woman given birth and a female birth attendant—is complex in shaping the micropolitics of the encounter, for example in how paternalistic biomedical systems of birth are	Feminist theories that focus attention on gender-based inequities will be more impactful when re-strategized to have analyses rooted in African contexts, such as Afro-feminism (Tamale) and Africana womanism (Hudson–Weems). Intersectionality, as a characteristic of feminist theorizing, is needed to capture the multifaceted nature of structural violence
3. Deficiency discourses are dominant in the included sources, foregrounding disadvantage, negativity, and failure while overlooking the historical, socioeconomic, and political structures that create and reinforce inequities. A deficiency approach overlooks people’s strengths and capabilities, including how individual and collective resilience is determined by connection to culture, community, and ancestry^16^	Strengths-based approaches are needed to identify protective factors already existing within individuals, communities, and systems, and how to amplify these. This approach does not deny inequities and structural violence; instead, it aims to shift the narrative and focus to identifying capabilities, assets, and strengths (i.e., protective factors) among individuals and groups; and identifying alternative mechanisms to address health issues (Islam)
4. Most albinism literature glosses over the pre-colonial mistreatment of PWA, the impact of colonial legacies, ongoing neocolonialism, and geopolitical inequities that require decolonization	Critical theorizing is needed about the impact of historical and global systems of power, including the decolonial reclaiming of protective Indigenous knowledges and moral systems (such as *Ubuntu*)
5. Human rights analyses tend to rely on universalizing international standards to protect the rights of individuals, an approach that has been criticized as not aligned with communitarian worldviews and the unique denial of the personhood or humanity of children born with albinism^90^	Legal theories need to emphasize the personhood and humanity of such groups when applying the human rights framework. Such endeavors should involve custodians of culture, spiritual practices and traditions. There is also a need to critically address aspects of communal values including the right to culture protected by the *African Charter on Human and Peoples’ Rights.* Some of these communal values effectively exclude groups like CWA for indicating a certain disharmony by their appearance. Communal values should in practice not derogate from individual rights which are equally protected under the Charter
6. Human rights literature has not adequately addressed what it means to have structural violations on human rights^127^	Given the extent to which structural violence denigrates the human rights of mothers and children impacted by albinism, special protection of social and economic rights, together with the right to development, should be equally emphasized as civil and political rights when considering albinism and human rights

CWA: child with albinism; RMC: respectful maternity care.

We identified several types of gaps in the existing literature: evidentiary, implementation, and theoretical. Evidentiary gaps are those that require empirical data and the generation of new knowledge, such as the gap on how strengths-based approaches can improve the birth experiences for mothers of a CWA, fathers’ experiences and roles, and long-term mental health effects. Implementation gaps are those situations in which sufficient evidence exists, but is not translated into practice, as with the example of the gaps in implementing human rights instruments. Theoretical gaps stem from the identification of conceptual apparatuses that could be well operationalized to further empirical and implementation efforts (i.e., a need for research and scholarship from critical perspectives that incorporate African epistemologies and tend to gender justice). Table 7 summarizes these gaps in knowledge (some of which are taken up in the next phases of our project) and suggests future research. Albinism research on human rights and psychosocial aspects of albinism in Africa is still a relatively new field. Based on our review, we recommend decolonizing Indigenous methodologies informed by strengths-based approaches and led by PWA and scholars in Africa in conjunction with intersectoral networks (see Table 7 for Topics).

### Protective factors to counter a matrix of violence (sense-giving)

An integrative review^126^ has as its intention a redirection, that is, “an alteration to the field’s perspective on a topic” (p. 173). We have attempted to make a compelling case for our interpretation of strengths-based responses to the birth of a CWA as resistance to the matrix of violence that sets a trajectory for precarity rather than flourishing. Our participatory method facilitated the validation of the co-joined central themes of community-based flourishing and structural violence.

A critique of a structural violence approach is that the agency and capacity of individuals can be overlooked or even silenced. “*Ubuntu* demands that we always keep the well-being of the collective in mind while never underestimating the value of individuality” (p. 68).^128^ For mothers and their children with albinism, their strengths and resistance to violence should be made more visible in research, practice, and policy.

Taking a capacity-oriented approach, while remaining responsive to how structural inequities and structural violence influence people’s health and well-being, is essential to enacting TVIC approaches. When awareness of structural conditions is foregrounded, service providers can acknowledge people’s strengths and resourcefulness while simultaneously seeking to understand, attend to, and affirm their capacities (p. 30).^129^

The good practices found in our review require government investment. Social inclusion and gender justice, respectful maternity care, a healthcare workforce with primary knowledge about the genetic cause of albinism, and the integration of Indigenous knowledges (including traditional birthing and *Ubuntu*) must be priorities to reduce the harm that families and their CWA endure in the perinatal period and beyond. Investment in the perinatal period will yield better outcomes and reduced expenditures in the long run.

*Obstetrical violence*, described by Davis-Floyd and Premkumar^130^ as the “darker side of biomedical maternity care,” involves the dehumanizing medicalization of birth and the suppression of Indigenous birthing epistemologies. Mogale^131^ pointed to “the urgent need to (re)write and (re)look at African and indigenous health care ways of knowing” (p. 2). Respectful Maternity Care^132^ has been posed as an antidote to obstetrical violence, though this framework also requires strengthening as to system influences and a decolonial lens.^7,133,134^ A trauma- and violence-informed framework reveals how the birth of a CWA is a trauma-event on account of its unexpectedness and the stigma, discrimination, and violence that follow (including *symbolic violence*). Along with family and community members, birth attendants too perpetuate prejudicial and discriminatory behavior that leads to inequities and harm, which requires recognition and management of the implicit biases.^135^ As an offspring of capitalism and colonialization,^136^ neoliberal economic globalization and the various forms of symbolic violence it produces has undermined Indigenous thought, epistemology, and ways of being.^137^ Accurate knowledge about albinism must be embedded through African epistemic systems,^138^ even as Africa cosmology constructs PWA as non-, sub-, or supra-human to protect communities from harm. *Ubuntu*, as a moral philosophy of humanness based on principles of care and community, harmony, hospitality, respect, and responsiveness, can provide broad guidance in countering structural violence and building justice-loving communal spaces, including in relation to *gender violence*.^128^ With colonial suppression of African knowledge systems came patriarchal, hierarchical, and dualistic worldviews about gender that subjugated African women. These are the conditions that prompted the South African legislation (*Domestic Violence Act* of 1998, and the *Criminal Law* on Sexual Offense and Related Matters of 2007) to protect women from intimate partner violence.^139^ Mogale et al.^139^ called for community engagement to strengthen support for gender equality and accountabilities for governments and judicial, health, and other sectors. Recent work, such as the volume on *Gender, African Philosophies, and Concepts* by Dube et al.,^140^ undergirds the way forward for women’s resilience and resistance for an inclusive, gender-just society.

With a systems view of *healthcare services* as experienced by mothers who give birth to a CWA, shortage of resources (such as access to specialized genetic, ophthalmology, and dermatology), crowded conditions and heavy workload of birth settings should be informed by SDG 3.8 Universal Health Coverage. Kipo-Sunyehzi’s^141^ review of health insurance policies in Rwanda, Tanzania, South Africa, and Ghana found that most African states have national or community-based health insurance schemes that cover about half of their populations, and African states tend to spend less than 10% of their Gross Domestic Product (GDP) on health (a slightly lower percentage than high-resource countries that have much higher GDPs). Designated funding for albinism-specific care (such as sunscreen and specialty services) for the relatively small population of PWA would go far.

Several recent systematic reviews of *health professions education* in Africa relate to our analysis.^142–149^ In the context of global health workforce shortages and Global North employers depleting the Global South workforce, health professions education faces perpetual educator shortage^143^ along with inadequate facilities and materials.^144^ In relation to midwifery education in sub-Saharan Africa, Warren et al.^148^ reported a misalignment between international standards for midwifery education and what local programs and larger administrative systems can reliably provide. Inadequate infrastructure, teaching capacity in school and clinical settings, and clinical site environment are barriers to education. Yet, there is innovation and robustness in current approaches, with curriculum re-design that foregrounds evidence-informed, community-based (primary healthcare), and interprofessional practice^149^ and moves toward decolonization in post-independence Africa.^150,151^ International investment and global partnerships^147,152^ are reported as strengthening health professions education.^153^ These developments create openings for inclusion of albinism-related education to better equip the health workforce to mitigate structural violence.

Regarding the *human rights* surrounding the birth of a baby with albinism, our review suggests that, as with other albinism-related human rights instruments, sufficient legal guidance and jurisprudence exists. Hence, the concern is more that of an implementation gap. An aspect of this gap is the vernacularization or contextualization of universal human rights to local cases, such as albinism in Africa. Ibhawoh et al.^90^ called for a counterhegemonic approach to human rights that goes beyond possessive individualism and the neoliberal, state-centered rights model. In a decolonial era, what is needed is a re-orientation to foreground community responsiveness. Tamale^47^ in her treatise on *Decolonization and Afro-Feminism* explained,

Under a communal inclusive society, rights are claims not against the state but society: . . .a social paradigm based on reciprocity, solidarity and inclusiveness—values that are far richer than the basis on which modern rights have been founded. . .culture is a key to justice (p. 128).

The notion of claims against society (not just the state) is not well explicated in the included literature, but is hinted at with many calls for public engagement and public education, as well as mothers’ resistance to not being treated well.

### Limitations

This review has several limitations worth noting. Despite considerable efforts to ensure a comprehensive search, relevant articles may have been missed due to the lack of controlled vocabulary related to albinism and the restriction to English literature. To maintain a clear focus, we limited our scope to articles published in Africa, which affects the applicability of findings elsewhere. While all included articles contributed to our synthesis, there was limited evidence primarily focusing on mothers’ and families’ experiences of birth and the first year of the child(ren)’s life, restricting our interpretation. Variability in study quality should also be considered when applying these findings. Finally, integrating diverse methodologies and article types posed challenges due to inconsistencies in purposes, study designs, and populations, which at times not only limited comparability but also provided a more comprehensive understanding.

## Conclusion

This participatory systematic review found a dearth of research that has as primary focus the perinatal experience of families and their carers at the birth of a CWA. The six primary studies (set in Tanzania, Malawi, Uganda, and South Africa) have analogous findings, which are supported by latent data from another 76 sources. The participatory process was invaluable for contextualizing the data to lived experiences in African contexts. By taking a trauma- and violence-informed lens, our analysis reveals the matrix of structural violence that characterizes the experience of mothers, and conversely, protective factors that become visible with a strengths-based framing. Our review findings show the promise of multi-pronged, multi-audience studies, and learning from missing voices (i.e., fathers, birth attendants, decision-makers) in the circle surrounding the birth of a CWA.

## Supplemental Material

sj-docx-1-whe-10.1177_17455057251395420 – Supplemental material for A participatory systematic review on human rights and the birth of a child with albinism in sub-Saharan Africa Supplemental material, sj-docx-1-whe-10.1177_17455057251395420 for A participatory systematic review on human rights and the birth of a child with albinism in sub-Saharan Africa by Sheryl Reimer-Kirkham, Kendra Rieger, Barbara Astle, Meghann Buyco, Kwame Andrews Daklo, Duncan Dixon, Ikponwosa Ero, Bonny Ibhawoh, Ingrid Tshegofatso Keitseomore, Jennifer Kromberg, Michael Lang, Ronell Leech, Nomasonto Mazibuko, Tumisho Mokwele, Tintswalo Victoria Nesengani, Lillian Ohene, Perpetua Senkoro, Eunice Siaity-Pallangyo, Landa Terblanche, Wisdom Tettey, Mpho Tjope, Lorraine Tshuma, Ingrid Watts, Jessica Wilson and Ramadimetja Shirley Mooa in Women's Health

sj-docx-2-whe-10.1177_17455057251395420 – Supplemental material for A participatory systematic review on human rights and the birth of a child with albinism in sub-Saharan Africa Supplemental material, sj-docx-2-whe-10.1177_17455057251395420 for A participatory systematic review on human rights and the birth of a child with albinism in sub-Saharan Africa by Sheryl Reimer-Kirkham, Kendra Rieger, Barbara Astle, Meghann Buyco, Kwame Andrews Daklo, Duncan Dixon, Ikponwosa Ero, Bonny Ibhawoh, Ingrid Tshegofatso Keitseomore, Jennifer Kromberg, Michael Lang, Ronell Leech, Nomasonto Mazibuko, Tumisho Mokwele, Tintswalo Victoria Nesengani, Lillian Ohene, Perpetua Senkoro, Eunice Siaity-Pallangyo, Landa Terblanche, Wisdom Tettey, Mpho Tjope, Lorraine Tshuma, Ingrid Watts, Jessica Wilson and Ramadimetja Shirley Mooa in Women's Health

sj-docx-3-whe-10.1177_17455057251395420 – Supplemental material for A participatory systematic review on human rights and the birth of a child with albinism in sub-Saharan Africa Supplemental material, sj-docx-3-whe-10.1177_17455057251395420 for A participatory systematic review on human rights and the birth of a child with albinism in sub-Saharan Africa by Sheryl Reimer-Kirkham, Kendra Rieger, Barbara Astle, Meghann Buyco, Kwame Andrews Daklo, Duncan Dixon, Ikponwosa Ero, Bonny Ibhawoh, Ingrid Tshegofatso Keitseomore, Jennifer Kromberg, Michael Lang, Ronell Leech, Nomasonto Mazibuko, Tumisho Mokwele, Tintswalo Victoria Nesengani, Lillian Ohene, Perpetua Senkoro, Eunice Siaity-Pallangyo, Landa Terblanche, Wisdom Tettey, Mpho Tjope, Lorraine Tshuma, Ingrid Watts, Jessica Wilson and Ramadimetja Shirley Mooa in Women's Health

sj-docx-4-whe-10.1177_17455057251395420 – Supplemental material for A participatory systematic review on human rights and the birth of a child with albinism in sub-Saharan Africa Supplemental material, sj-docx-4-whe-10.1177_17455057251395420 for A participatory systematic review on human rights and the birth of a child with albinism in sub-Saharan Africa by Sheryl Reimer-Kirkham, Kendra Rieger, Barbara Astle, Meghann Buyco, Kwame Andrews Daklo, Duncan Dixon, Ikponwosa Ero, Bonny Ibhawoh, Ingrid Tshegofatso Keitseomore, Jennifer Kromberg, Michael Lang, Ronell Leech, Nomasonto Mazibuko, Tumisho Mokwele, Tintswalo Victoria Nesengani, Lillian Ohene, Perpetua Senkoro, Eunice Siaity-Pallangyo, Landa Terblanche, Wisdom Tettey, Mpho Tjope, Lorraine Tshuma, Ingrid Watts, Jessica Wilson and Ramadimetja Shirley Mooa in Women's Health

sj-docx-5-whe-10.1177_17455057251395420 – Supplemental material for A participatory systematic review on human rights and the birth of a child with albinism in sub-Saharan Africa Supplemental material, sj-docx-5-whe-10.1177_17455057251395420 for A participatory systematic review on human rights and the birth of a child with albinism in sub-Saharan Africa by Sheryl Reimer-Kirkham, Kendra Rieger, Barbara Astle, Meghann Buyco, Kwame Andrews Daklo, Duncan Dixon, Ikponwosa Ero, Bonny Ibhawoh, Ingrid Tshegofatso Keitseomore, Jennifer Kromberg, Michael Lang, Ronell Leech, Nomasonto Mazibuko, Tumisho Mokwele, Tintswalo Victoria Nesengani, Lillian Ohene, Perpetua Senkoro, Eunice Siaity-Pallangyo, Landa Terblanche, Wisdom Tettey, Mpho Tjope, Lorraine Tshuma, Ingrid Watts, Jessica Wilson and Ramadimetja Shirley Mooa in Women's Health

sj-docx-6-whe-10.1177_17455057251395420 – Supplemental material for A participatory systematic review on human rights and the birth of a child with albinism in sub-Saharan Africa Supplemental material, sj-docx-6-whe-10.1177_17455057251395420 for A participatory systematic review on human rights and the birth of a child with albinism in sub-Saharan Africa by Sheryl Reimer-Kirkham, Kendra Rieger, Barbara Astle, Meghann Buyco, Kwame Andrews Daklo, Duncan Dixon, Ikponwosa Ero, Bonny Ibhawoh, Ingrid Tshegofatso Keitseomore, Jennifer Kromberg, Michael Lang, Ronell Leech, Nomasonto Mazibuko, Tumisho Mokwele, Tintswalo Victoria Nesengani, Lillian Ohene, Perpetua Senkoro, Eunice Siaity-Pallangyo, Landa Terblanche, Wisdom Tettey, Mpho Tjope, Lorraine Tshuma, Ingrid Watts, Jessica Wilson and Ramadimetja Shirley Mooa in Women's Health

sj-docx-7-whe-10.1177_17455057251395420 – Supplemental material for A participatory systematic review on human rights and the birth of a child with albinism in sub-Saharan Africa Supplemental material, sj-docx-7-whe-10.1177_17455057251395420 for A participatory systematic review on human rights and the birth of a child with albinism in sub-Saharan Africa by Sheryl Reimer-Kirkham, Kendra Rieger, Barbara Astle, Meghann Buyco, Kwame Andrews Daklo, Duncan Dixon, Ikponwosa Ero, Bonny Ibhawoh, Ingrid Tshegofatso Keitseomore, Jennifer Kromberg, Michael Lang, Ronell Leech, Nomasonto Mazibuko, Tumisho Mokwele, Tintswalo Victoria Nesengani, Lillian Ohene, Perpetua Senkoro, Eunice Siaity-Pallangyo, Landa Terblanche, Wisdom Tettey, Mpho Tjope, Lorraine Tshuma, Ingrid Watts, Jessica Wilson and Ramadimetja Shirley Mooa in Women's Health
