# Supplementary material for: A participatory systematic review on human rights and the birth of a child with albinism in sub-Saharan Africa
Source: Womens Health (Lond). 2025 Dec 11;21:17455057251395420. doi: 10.1177/17455057251395420 (PMC12699010; doi:10.1177/17455057251395420)
Supplement: sj-docx-1-whe-10.1177_17455057251395420 – Supplemental material for A participatory systematic review on human rights and the birth of a child with albinism in sub-Saharan Africa [file sj-docx-1-whe-10.1177_17455057251395420.docx]

**Supplemental File: Search Strings for Academic Databases**

| **CONCEPT #1:**  Sub-Saharan Africa | **AND** | **CONCEPT #2:**  Albinism |  | **CONCEPT #3:**  Birth Attendants | **EITHER 3 OR 4** | **CONCEPT #4:**  Perinatal |
| --- | --- | --- | --- | --- | --- | --- |

**For all databases, no limiters or filters were applied.**

1. **Academic Search Complete:**
2. (DE "SUB-Saharan African people") OR (DE "SOUTHERN African literature") OR (DE "ANGOLA in literature") OR (DE "ANGOLAN literature") OR (DE "BENINESE literature") OR (DE "BOTSWANA in literature") OR (DE "BOTSWANANS") OR (DE "BURUNDIANS") OR (DE "CAMEROONIAN literature") OR (DE "CAMEROONIAN literature (English)”) OR (DE "CAMEROONIAN literature (French)") OR (DE "CAMEROONIANS") OR (DE "CENTRAL African (Central African Republic) literature") OR (DE "CENTRAL Africans (Central African Republic)") OR (DE "CHADIAN literature") OR (DE "COMORIANS") OR (DE "KONGO (African people)") OR (DE "CONGOLESE (Brazzaville) literature") OR (DE "CONGOLESE (Brazzaville) literature (French)") OR (DE "IVOIRIANS") OR (DE "EQUATORIAL Guinean literature") OR (DE "EQUATORIAL Guineans") OR (DE "ERITREAN literature") OR (DE "ERITREANS") OR (DE "ETHIOPIA in literature") OR (DE "GABONESE") OR (DE "GABONESE literature") OR (DE "GABONESE literature (French)") OR (DE "GAMBIAN literature") OR (DE "GAMBIANS") OR (DE "GHANAIAN literature") OR (DE "GUINEANS (Guinea-Bissauans)") OR (DE "KENYAN literature") OR (DE "LIBERIA in literature") OR (DE "LIBERIAN literature") OR (DE "LIBERIANS") OR (DE "MALAWI literature") OR DE "MALAWIANS") OR (DE "MALIAN literature") OR (DE "MALIANS") OR (DE "MAURITANIAN literature") OR (DE "MAURITANIANS") OR (DE "MAURITIAN literature") OR (DE "MAURITIAN literature (French)") OR (DE "MOZAMBIQUE in literature") OR (DE "NAMIBIAN literature") OR (DE "NAMIBIANS") OR (DE "NIGERIA in literature") OR (DE "REUNIONESE") OR (DE "REUNIONESE literature (French)") OR (DE "RWANDAN literature (French)") OR (DE "RWANDANS") OR (DE "SENEGAL in literature") OR (DE "SENEGALESE") OR (DE "SOMALIA in literature") OR (DE "SOUTH Africa in literature") OR (DE "SUDANESE") OR (DE "TANZANIAN literature") OR (DE "TANZANIANS") OR (DE "TOGOLESE literature") OR (DE "UGANDA in literature") OR (DE "UGANDAN literature") OR (DE "UGANDAN literature (English)") OR (DE "ZAMBIAN literature") OR (DE "ZAMBIANS") OR (DE "ZIMBABWE in literature") OR (DE "ZIMBABWEAN literature") OR DE "ZIMBABWEAN literature (English)" OR (DE "ZIMBABWEANS") OR Africa* OR "sub-Saharan" OR Angola OR Benin OR Botswana OR "Burkina Faso" OR Burundi OR Cameroon OR "Cape Verde" OR "Central African Republic" OR Chad OR Comoros OR Congo OR "Côte d'Ivoire" OR "Ivory Coast" OR Djibouti OR "Equatorial Guinea" OR Eritrea OR Ethiopia OR Gabon OR "The Gambia" OR Ghana OR Ghanian OR Guinea OR "Guinea-Bissau" OR Kenya OR Lesotho OR Liberia OR Madagascar OR Malawi* OR Mali OR Mauritania OR Mauritius OR Mozambique OR Namibia OR Niger OR Nigeria OR Réunion OR Rwanda OR "Sao Tome and Principe" OR Senegal OR Seychelles OR "Sierra Leone" OR Somalia OR "South Africa*" OR Sudan OR Swaziland OR Tanzania OR Togo OR Uganda OR "Western Sahara" OR Zambia OR Zimbabwe
3. (DE "ALBINOS & albinism") OR Albinism OR Albino* OR achrom* OR muzungu OR oculocutaneous OR hypopigment*
4. DE "AUNTS" OR DE "AUNTS in literature" OR DE "ANESTHESIOLOGIST assistants" OR DE "ANESTHESIOLOGISTS" OR DE "ANESTHESIOLOGY" OR DE "COUNSELORS" OR DE "BLACK nurses" OR DE "BROTHERS" OR DE "BROTHERS in literature" OR DE "COUSINS" OR DE "DERMATOLOGISTS" OR DE "DERMATOLOGY" OR DE "FAMILIES" OR DE "PATIENT-family relations" OR DE "FATHER-child relationship" OR DE "FATHER-child relationship in literature" OR DE "FATHER-daughter relationship" OR DE "FATHER-daughter relationship in literature" OR DE "FATHER-infant relationship" OR DE "FATHER-son relationship" OR DE "FATHER-son relationship in literature" OR DE "FATHER-son relationship in motion pictures" OR DE "FATHERHOOD" OR DE "FATHERHOOD in literature" OR DE "FATHERLESS families" OR DE "FATHERLESS families in literature" OR DE "FATHERS" OR DE "FATHERS in literature" OR DE "FATHERS-in-law" OR DE "GRANDMOTHERS" OR DE "GRANDMOTHERS in literature" OR DE "GRANDPARENT-grandchild relationships" OR DE "GRANDPARENTING" OR DE "GRANDPARENTS" OR DE "GYNECOLOGIST & patient" OR DE "GYNECOLOGISTS" OR DE "GYNECOLOGY" OR DE "HEALERS" OR DE "HEALTH services accessibility" OR DE "HUSBAND & wife" OR DE "HUSBAND & wife (Canon law)" OR DE "HUSBANDS" OR DE "HUSBANDS in literature" OR DE "MAMA (African people)" OR DE "MEDICAL care" OR DE "MEDICAL care & globalization" OR DE "MIDWIFERY" OR DE "MIDWIFERY in literature" OR DE "MIDWIVES" OR DE "MIDWIVES in literature" OR DE "MOTHER-daughter relationship in literature" OR DE "MOTHER-infant relationship" OR DE "MOTHER-son relationship" OR DE "MOTHER-son relationship in literature" OR DE "MOTHERHOOD" OR DE "MOTHERS" OR DE "MOTHERS in literature" OR DE "MOTHERS-in-law" OR DE "MOTHERS-in-law & daughters-in-law" OR DE "MOTHERS-in-law in literature" OR DE "NEONATOLOGISTS" OR DE "NEONATOLOGY" OR DE "NURSE anesthetists" OR DE "NURSE practitioners" OR DE "NURSE training" OR DE "NURSE-patient relationships" OR DE "NURSE-physician relationships" OR DE "NURSES" OR DE "NURSES in literature" OR DE "NURSING" OR DE "NURSING & society" OR DE "NURSING care facilities" OR DE "OBSTETRICIANS" OR DE "OBSTETRICS" OR DE "OPHTHALMOLOGISTS" OR DE "OPHTHALMOLOGY" OR DE "PEDIATRICIANS" OR DE "PEDIATRICS" OR DE "PHYSICIAN training" OR DE "HOSPITAL-physician relations" OR DE "HOSPITALISTS" OR DE "PHYSICIAN & patient in literature" OR DE "PHYSICIAN-patient relations" OR DE "PHYSICIANS" OR DE "RELATIVES" OR DE "SIBLING attachment" OR DE "SIBLINGS" OR DE "SIBLINGS in literature" OR DE "SIBLINGS of people with disabilities" OR DE "SISTERS" OR DE "SISTERS in literature" OR DE "SISTERS-in-law" OR DE "NURSING students" OR DE "SOCIAL worker & client" OR DE "SOCIAL workers" OR DE "AFRICAN traditional medicine" OR DE "TRADITIONAL medicine" OR DE "WOMEN" OR Aunt* OR anesthesiologist* OR anaesthesiologist* OR "birth attendant*" OR Brother* OR carer* OR companion* OR counsellor OR counselor OR Cousin OR Dermatologist OR docto* OR famil* OR father* OR grandmother* OR gynecologist OR gynaecologist OR healer* OR healthcare OR "health care" OR husband* OR "in-law*" OR Mama* OR midwi* OR Mother* OR neonatologist* OR nurs* OR obstetrician* OR Ophthalmologist OR Paediatrician OR Pediatrician OR partner* OR physician* OR relative* OR "RN" OR sibling* OR sister* OR "social work*" OR student* OR TBA OR tradition* OR woman OR women
5. DE "BREASTFEEDING" OR DE "BREASTFEEDING in literature" OR DE "CESAREAN section" OR DE "CESAREAN section nursing" OR DE "CHILDBIRTH" OR DE "CHILDBIRTH & psychology" OR DE "DELIVERY (Obstetrics)" OR DE "HORMONAL aspects of labor (Obstetrics)" OR DE "INTRAPARTUM care" OR DE "LABOR (Obstetrics)" OR DE "MATERNITY nursing" OR DE "MATERNAL love" OR DE "MATERNAL rejection" OR DE "MATERNAL-child health services" OR DE "MOTHERHOOD" OR DE "CHILDBIRTH at home" OR DE "RURAL health" OR DE "RURAL health clinics" OR DE "RURAL health services" OR DE "RURAL hospitals" OR DE "URBAN health" OR DE "MATERNAL & infant welfare" OR DE "MATERNAL health" OR DE "MATERNAL health care teams" OR DE "MATERNAL health services" OR DE "MULTIPARAS" OR DE "NEWBORN infants -- Nutrition" OR DE "NEWBORN infants -- Psychology" OR DE "NEWBORN infant care" OR DE "NEWBORN infants" OR DE "VAGINAL birth after cesarean" OR DE "PERINATAL growth" OR DE "PERINATAL period" OR DE "PERINEAL care" OR DE "PREGNANCY" OR DE "PREGNANCY -- Psychological aspects" OR DE "PRENATAL care" OR DE "PRIMIPARAS" OR DE "PUERPERIUM" OR DE "POSTNATAL care" OR DE "POSTOPERATIVE care" OR antepartum OR ante-partum OR antenatal OR "ante-natal" OR baby OR birth* OR caesarean OR cesarean OR "c-section" OR childbearing OR "child-bearing" OR Childbirth OR Deliver* OR gestation OR infant OR intrapartum OR "intra-partum" OR "labour and delivery" OR "labor and delivery" OR maternal OR Maternity OR multigravida OR "multi-gravida" OR multipara OR "multi-para" OR multiparous OR "multi-parous" OR neonate OR "neo-nate" OR neonatal OR "neo-natal" OR newborn OR "new-born" OR obstetric* OR perinatal OR "peri-natal" OR postnatal OR "post-natal" OR postpartum OR "post-partum" OR pregnan* OR prenatal OR "pre-natal" OR primipara OR primiparous OR primigravida OR puerperium OR puerperal OR womb
6. 1 AND 2 AND 3
7. 1 AND 2 AND 4
8. **CINAHL**
9. (MH "Africa South of the Sahara+") OR Africa* OR "sub-Saharan" OR Angola OR Benin OR Botswana OR "Burkina Faso" OR Burundi OR Cameroon OR "Cape Verde" OR "Central African Republic" OR Chad OR Comoros OR Congo OR "Côte d'Ivoire" OR "Ivory Coast" OR Djibouti OR "Equatorial Guinea" OR Eritrea OR Ethiopia OR Gabon OR "The Gambia" OR Ghana OR Ghanian OR Guinea OR "Guinea-Bissau" OR Kenya OR Lesotho OR Liberia OR Madagascar OR Malawi* OR Mali OR Mauritania OR Mauritius OR Mozambique OR Namibia OR Niger OR Nigeria OR Réunion OR Rwanda OR "Sao Tome and Principe" OR Senegal OR Seychelles OR "Sierra Leone" OR Somalia OR "South Africa*" OR Sudan OR Swaziland OR Tanzania OR Togo OR Uganda OR "Western Sahara" OR Zambia OR Zimbabwe
10. (MH "Albinism") OR (MH "Hypopigmentation+") OR Albinism OR Albino* OR achrom* OR muzungu OR oculocutaneous OR hypopigment*
11. (MH "Lay Midwives") OR (MH "Nurse Midwives") OR (MH "Midwives+") OR (MH "Students, Midwifery") OR (MH "Nurse Counselors") OR (MH "Counselors+") OR (MH "Psychotherapist Attitudes") OR (MH "Family+") OR (MH "Extended Family+") OR (MH "Family Attitudes+") OR (MH "Family Services") OR (MH "Family Nursing") OR (MH "Patient-Family Conferences") OR (MH "Patient-Family Relations") OR (MH "Family Relations+") OR (MH "Professional-Family Relations") OR (MH "Physicians, Family") OR (MH "Anesthesiologists") OR (MH "Dermatologists") OR (MH "Traditional Healers") OR (MH "Spouses") OR (MH "Mothers+") OR (MH "Parents with Disabilities") OR (MH "Parents of Children with Disabilities") OR (MH "Parents+") OR (MH "Fathers+") OR (MH "Expectant Parents+") OR (MH "Mother-Infant Relations") OR (MH "Adolescent Mothers") OR (MH "Mother-Child Relations") OR (MH "Expectant Mothers") OR (MH "Neonatologists") OR (MH "Nurses+") OR (MH "Ophthalmologists") OR (MH "Ophthalmic Technologists") OR (MH "Pediatricians") OR (MH "Physicians+") OR (MH "Siblings") OR (MH "Social Workers") OR (MH "Social Worker Attitudes") OR (MH "Women+") OR (MH "Lay Midwifery") OR (MH "Adolescent Fathers") OR (MH "Adolescent Mothers") OR (MH "Charge Nurses") OR (MH "Childbirth Educators") OR (MH "Counselors") OR (MH "Emergency Nurse Practitioners") OR (MH "Expectant Fathers") OR (MH "Family") OR (MH "Family Nurse Practitioners") OR (MH "Family Relations") OR (MH "Father-Infant Relations") OR (MH "Father-Child Relations") OR (MH "Grandparents") OR (MH "Head Nurses") OR (MH "Health Personnel") OR (MH "Married Women") OR (MH "Maternal Age 35 and Over") OR (MH "Maternal-Child Care") OR (MH "Maternal-Child Health") OR (MH "Maternal-Child Nursing") OR (MH "Maternal-Child Welfare") OR (MH "Medical Staff") OR (MH "Medical Staff, Hospital") OR (MH "Medicine, African Traditional") OR (MH "Midwifery Service") OR (MH "Mother-Child Relations") OR (MH "Mother-Infant Relations") OR (MH "Motherhood") OR (MH "Mothers") OR (MH "Multidisciplinary Care Team") OR (MH "Neonatal Nurse Practitioners") OR (MH "Neonatal Nursing") OR (MH "Multidisciplinary Care Team") OR (MH "Neonatal Nurse Practitioners") OR (MH "Neonatal Nursing") OR (MH "Neonatologists") OR (MH "Nuclear Family") OR (MH "Nurse Counselors") OR (MH "Nurse Consultants") OR (MH "Nurse Liaison") OR (MH "Nurse Managers") OR (MH "Nurse Midwifery") OR (MH "Nurse Midwives") OR (MH "Nurse Practitioners") OR (MH "Nurses") OR (MH "Nurses, Male") OR (MH "Nursing Assistants") OR (MH "Nursing Staff, Hospital") OR (MH "Ophthalmologists") OR (MH "Parents") OR (MH "Pediatric Nurse Practitioners") OR (MH "Perinatal Nursing") OR (MH "Pediatric Nursing") OR (MH "Physicians, Family") OR (MH "Physicians, Women") OR (MH "Physicians, Emergency") OR (MH "Physician Assistants") OR (MH "Psychiatrists") OR (MH "Physician-Patient Relations") OR (MH "Practical Nursing") OR (MH "Practical Nurses") OR (MH "Quality of Nursing Care") OR (MH "Registered Nurses") OR (MH "RN First Assistants") OR (MH "Rural Health Personnel") OR (MH "Single Parent") OR (MH "Single Women") OR (MH "Social Work") OR (MH "Staff Nurses") OR (MH "Students, Midwifery") OR (MH "Students, Nurse Midwifery") OR (MH "Students, Nursing, Doctoral") OR (MH "Students, Nursing, Male") OR (MH "Students, Nursing, Graduate") OR (MH "Students, Nursing, Associate") OR (MH "Students, Nursing") OR (MH "Students, Nursing, Masters") OR (MH "Students, Nursing, Baccalaureate") OR (MH "Students, Physician Assistant") OR (MH "Students, Medical") OR (MH "Students") OR (MH "Physicians") OR (MH "Spouses") OR (MH "Traditional Healers") OR (MH "Women") OR (MH "Women's Health") OR (MH "Women's Health Services") OR (MH "Women's Rights") OR (MH "Women, Working") OR Aunt* OR Anesthesiologist* OR anaesthesiologist OR "birth attendant*" OR Brother* OR carer* OR companion* OR counsellor* OR counselor* OR Cousin* OR Dermatologist* OR docto* OR famil* OR father* OR grandmother* OR gynecologist* OR gynaecologist* OR healer* OR healthcare OR "health care" OR husband* OR "in-law*" OR Mama* OR midwi* OR Mother* OR neonatologist* OR nurs* OR obstetrician* OR Ophthalmologist* OR Paediatrician* OR Pediatrician* OR partner* OR physician* OR relative* OR "RN*" OR sibling* OR sister* OR "social work*" OR student* OR TBA* OR tradition* OR woman OR women
12. (MH "Prenatal Care") OR (MH "Infant, Newborn, Diseases+") OR (MH "Infant Care+") OR (MH "Infant, Newborn+") OR (MH "Infant, High Risk") OR (MH "Mother-Infant Relations") OR (MH "Alternative Birth Centers") OR (MH "Infant, Low Birth Weight+") OR (MH "Term Birth") OR (MH "Birth Setting") OR (MH "Cesarean Section, Elective") OR (MH "Cesarean Section+") OR (MH "Cesarean Section, Repeat") OR (MH "Perinatal Nursing") OR (MH "Perinatal Care") OR (MH "Childbirth+") OR (MH "Prepared Childbirth") OR (MH "Home Childbirth") OR (MH "Childbirth, Premature") OR (MH "Childbirth Educators") OR (MH "Childbirth Education") OR (MH "Alternative Birth Methods+") OR (MH "Health Care Delivery") OR (MH "Nursing Care Delivery Systems") OR (MH "Delivery Rooms") OR (MH "Infant, Small for Gestational Age") OR (MH "Infant, Large for Gestational Age") OR (MH "Gestational Age") OR (MH "Infant+") OR (MH "Intrapartum Care") OR (MH "Maternal Behavior") OR (MH "Maternal Attitudes") OR (MH "Maternal-Child Welfare") OR (MH "Maternal-Child Nursing") OR (MH "Maternal-Child Care") OR (MH "Maternal-Child Health") OR (MH "Maternal Role") OR (MH "Maternal Health Services") OR (MH "Delivery, Obstetric+") OR (MH "Obstetric Patients") OR (MH "Obstetric Service") OR (MH "Diagnosis, Obstetric+") OR (MH "Obstetric Nursing") OR (MH "Obstetric Care+") OR (MH "Perinatal Period") OR (MH "Postnatal Period+") OR (MH "Postnatal Care+") OR (MH "Pregnancy+") OR (MH "Attitude to Pregnancy") OR (MH "Prenatal Diagnosis") OR (MH "Prenatal Bonding") OR (MH "Primiparas") OR (MH "Puerperium") OR (MH "Puerperal Disorders+") OR (MH "Uterus+") OR (MH "Prenatal Care") OR (MH "Infant, Hospitalized") OR (MH "Parent-Infant Bonding") OR (MH "Infant Feeding") OR (MH "Infant Care") OR (MH "Infant, Newborn") OR (MH "Infant, Premature") OR (MH "Infant, Postmature") OR (MH "Infant Care (Iowa NIC)") OR (MH "Father-Infant Relations") OR (MH "Newborn Care (Saba CCC)") OR (MH "Infant Care (Saba CCC)") OR (MH "Infant") OR (MH "Term Birth") OR (MH "Neonatal Nursing") OR (MH "Vaginal Birth") OR (MH "Vaginal Birth After Cesarean") OR (MH "Prenatal Care (Iowa NIC)") OR (MH "Pregnancy (Omaha)") OR (MH "Maternal-Child Welfare") OR (MH "Maternal-Child Nursing") OR (MH "Maternal-Child Care") OR (MH "Maternal-Child Health") OR (MH "Latching, Breastfeeding") OR (MH "Labor") OR (MH "Mother-Child Relations") OR (MH "Childbirth") OR (MH "Childbirth, Premature") OR (MH "Cesarean Section Care (Iowa NIC)") OR (MH "Cesarean Section") OR (MH "Breast Feeding") OR (MH "Birthing (Iowa NIC)") OR (MH "Mother-Infant Relations") OR (MH "Infant, Newborn, Diseases") OR (MH "Parent Education: Childbearing Family (Iowa NIC)") OR (MH "Childbearing Care (Iowa NIC)") OR (MH "Perinatal Nursing") OR (MH "Perinatal Care") OR (MH "Prepared Childbirth") OR (MH "Water Birth") OR (MH "Postnatal Period") OR (MH "Lactation") OR (MH "Labor Pain") OR (MH "Delivery Rooms") OR (MH "Health Care Delivery") OR (MH "Health Care Delivery, Integrated") OR (MH "Cesarean Section, Elective") OR (MH "Home Childbirth") OR (MH "Postpartum Care (Saba CCC)") OR (MH "Postnatal Care") OR (MH "Obstetric Care") OR (MH "Newborn Monitoring (Iowa NIC)") OR (MH "Labor Stage, Third") OR (MH "Intrapartum Care") OR (MH "Maternal Health Services") OR (MH "Pregnancy") OR (MH "Hospital Units+") OR (MM "Hospitals, Pediatric") OR (MM "Hospitals, Community") OR (MM "Hospitals") OR antepartum OR ante-partum OR antenatal OR "ante-natal" OR baby OR babies OR birth* OR caesarean OR cesarean OR "c-section" OR childbearing OR "child-bearing" OR Childbirth OR Deliver* OR gestation OR infant* OR intrapartum OR "intra-partum" OR "labour and delivery" OR "labor and delivery" OR maternal OR Maternity OR multigravida OR "multi-gravida" OR multipara OR "multi-para" OR multiparous OR "multi-parous" OR neonate OR "neo-nate" OR neonatal OR "neo-natal" OR newborn* OR "new-born*" OR obstetric* OR perinatal OR "peri-natal" OR postnatal OR "post-natal" OR postpartum OR "post-partum" OR pregnan* OR prenatal OR "pre-natal" OR primipara OR primiparous OR primigravida OR puerperium OR puerperal OR womb
13. 1 AND 2 AND 3
14. 1 AND 2 AND 4
15. **JSTOR**
16. Africa* OR "sub-Saharan" OR Angola OR Benin OR Botswana OR "Burkina Faso" OR Burundi OR Cameroon OR "Cape Verde" OR "Central African Republic" OR Chad OR Comoros OR Congo OR "Côte d'Ivoire" OR "Ivory Coast" OR Djibouti OR "Equatorial Guinea" OR Eritrea OR Ethiopia OR Gabon OR "The Gambia" OR Ghana OR Ghanian OR Guinea OR "Guinea-Bissau" OR Kenya OR Lesotho OR Liberia OR Madagascar OR Malawi* OR Mali OR Mauritania OR Mauritius OR Mozambique OR Namibia OR Niger OR Nigeria OR Réunion OR Rwanda OR "Sao Tome and Principe" OR Senegal OR Seychelles OR "Sierra Leone" OR Somalia OR "South Africa*" OR Sudan OR Swaziland OR Tanzania OR Togo OR Uganda OR "Western Sahara" OR Zambia OR Zimbabwe
17. Albinism OR Albino* OR achrom* OR muzungu OR oculocutaneous OR hypopigment*
18. 1 AND 2
19. **MEDLINE**
20. (MH "Africa South of the Sahara+") OR (MH "Africa, Central+") OR (MH "Africa, Eastern+") OR (MH "Africa, Southern+") OR (MH "Africa, Western+") OR (MH "Sub-Saharan African People") OR Africa* OR "sub-Saharan" OR Angola OR Benin OR Botswana OR "Burkina Faso" OR Burundi OR Cameroon OR "Cape Verde" OR "Central African Republic" OR Chad OR Comoros OR Congo OR "Côte d'Ivoire" OR "Ivory Coast" OR Djibouti OR "Equatorial Guinea" OR Eritrea OR Ethiopia OR Gabon OR "The Gambia" OR Ghana OR Ghanian OR Guinea OR "Guinea-Bissau" OR Kenya OR Lesotho OR Liberia OR Madagascar OR Malawi* OR Mali OR Mauritania OR Mauritius OR Mozambique OR Namibia OR Niger OR Nigeria OR Réunion OR Rwanda OR "Sao Tome and Principe" OR Senegal OR Seychelles OR "Sierra Leone" OR Somalia OR "South Africa*" OR Sudan OR Swaziland OR Tanzania OR Togo OR Uganda OR "Western Sahara" OR Zambia OR Zimbabwe
21. (MH "Albinism+") OR (MH "Albinism, Oculocutaneous+") OR (MH "Albinism, Ocular") OR (MH "Hypopigmentation+") OR Albinism OR Albino* OR achrom* OR muzungu OR oculocutaneous OR hypopigment*
22. (MH "Anesthesiologists") OR (MH "Anesthetists+") OR (MH "Nurse Anesthetists") OR (MH "Midwifery") OR (MH "Siblings") OR (MH "Caregivers") OR (MH "Friends") OR (MH "Women+") OR (MH "Volunteers+") OR (MH "Visitors to Patients") OR (MH "Students") OR (MH "Spouses") OR (MH "Sexual Partners") OR (MH "Parents+") OR (MH "Grandparents") OR (MH "Counselors") OR (MH "Dermatologists") OR (MH "Physicians+") OR (MH "Physicians, Women") OR (MH "Physicians, Family") OR (MH "Physicians, Primary Care") OR (MH "Physician Assistants") OR (MH "Medical Staff, Hospital+") OR (MH "General Practitioners") OR (MH "Family+") OR (MH "Fathers+") OR (MH "Gynecologists") OR (MH "Traditional Medicine Practitioners") OR (MH "Community Health Services") OR (MH "Health Services") OR (MH "Women's Health Services") OR (MH "Child Health Services") OR (MH "Nurse Midwives") OR (MH "Mothers+") OR (MH "Neonatologists") OR (MH "Nurses+") OR (MH "Nursing Staff+") OR (MH "Personnel, Hospital+") OR (MH "Optometrists") OR (MH "Nurses, Pediatric") OR (MH "Nurses, Neonatal") OR (MH "Nurses, Male") OR (MH "Nurses, Community Health") OR (MH "Licensed Practical Nurses") OR (MH "Nurses, Public Health") OR (MH "Nursing Assistants") OR (MH "Obstetricians") OR (MH "Ophthalmologists") OR (MH "Ophthalmic Assistants") OR (MH "Pediatricians") OR (MH "Nuns") OR (MH "Social Workers") OR (MH "Students, Nursing") OR (MH "Students, Premedical") OR (MH "Students, Medical") OR (MH "Students, Public Health") OR (MH "Students, Health Occupations") OR (MH "Medicine, African Traditional") OR (MH "Female") OR (MH "Lay Midwives") OR (MH "Nurse Midwives") OR (MH "Midwives+") OR (MH "Students, Midwifery") OR (MH "Nurse Counselors") OR (MH "Counselors+") OR (MH "Psychotherapist Attitudes") OR (MH "Family+") OR (MH "Extended Family+") OR (MH "Family Attitudes+") OR (MH "Family Services") OR (MH "Family Nursing") OR (MH "Patient-Family Conferences") OR (MH "Patient-Family Relations") OR (MH "Family Relations+") OR (MH "Professional-Family Relations") OR (MH "Physicians, Family") OR (MH "Anesthesiologists") OR (MH "Dermatologists") OR (MH "Traditional Healers") OR (MH "Spouses") OR (MH "Mothers+") OR (MH "Parents with Disabilities") OR (MH "Parents of Children with Disabilities") OR (MH "Parents+") OR (MH "Fathers+") OR (MH "Expectant Parents+") OR (MH "Mother-Infant Relations") OR (MH "Adolescent Mothers") OR (MH "Mother-Child Relations") OR (MH "Expectant Mothers") OR (MH "Neonatologists") OR (MH "Nurses+") OR (MH "Ophthalmologists") OR (MH "Ophthalmic Technologists") OR (MH "Pediatricians") OR (MH "Physicians+") OR (MH "Siblings") OR (MH "Social Workers") OR (MH "Social Worker Attitudes") OR (MH "Women+") OR (MH "Lay Midwifery") OR Aunt* OR Anesthesiologist* OR "birth attendant*" OR Brother* OR carer* OR companion* OR counsellor* OR counselor* OR Cousin* OR Dermatologist* OR docto* OR famil* OR father* OR grandmother* OR gynecologist* OR gynaecologist* OR healer* OR healthcare OR "health care" OR husband* OR "in-law*" OR Mama* OR midwi* OR Mother* OR neonatologist* OR nurs* OR obstetrician* OR Ophthalmologist* OR Paediatrician* OR Pediatrician* OR partner* OR physician* OR relative* OR "RN" OR sibling* OR sister* OR "social work*" OR student* OR TBA OR tradition* OR woman OR women
23. (MH "Prenatal Education") OR (MH "Prenatal Diagnosis") OR (MH "Prenatal Care") OR (MH "Infant+") OR (MH "Infant, Newborn+") OR (MH "Infant Behavior") OR (MH "Infant, Postmature") OR (MH "Infant Welfare") OR (MH "Nurseries, Infant") OR (MH "Infant, Premature") OR (MH "Infant Health") OR (MH "Parturition+") OR (MH "Birthing Centers") OR (MH "Term Birth") OR (MH "Premature Birth") OR (MH "Birth Setting+") OR (MH "Cesarean Section+") OR (MH "Reproductive Behavior+") OR (MH "Home Childbirth") OR (MH "Natural Childbirth") OR (MH "Delivery, Obstetric") OR (MH "Delivery Rooms") OR (MH "Pregnancy+") OR (MH "Pregnancy Outcome") OR (MH "Pregnancy Maintenance") OR (MH "Pregnancy Complications") OR (MH "Labor, Obstetric+") OR (MH "Maternal Behavior+") OR (MH "Maternal Welfare") OR (MH "Maternal Health") OR (MH "Maternal Health Services+") OR (MH "Maternal-Child Nursing+") OR (MH "Maternal-Fetal Relations") OR (MH "Mothers+") OR (MH "Adolescent Mothers") OR (MH "Gravidity") OR (MH "Postpartum Period") OR (MH "Parity") OR (MH "Peripartum Period") OR (MH "Obstetrics") OR (MH "Perinatal Care+") OR (MH "Postnatal Care") OR (MH "Uterus") OR antepartum OR ante-partum OR antenatal OR "ante-natal" OR baby OR birth* OR caesarean OR cesarean OR "c-section" OR childbearing OR "child-bearing" OR Childbirth OR Deliver* OR gestation OR infant OR intrapartum OR "intra-partum" OR "labour and delivery" OR "labor and delivery" OR maternal OR Maternity OR multigravida OR "multi-gravida" OR multipara OR "multi-para" OR multiparous OR "multi-parous" OR neonate OR "neo-nate" OR neonatal OR "neo-natal" OR newborn OR "new-born" OR obstetric* OR perinatal OR "peri-natal" OR postnatal OR "post-natal" OR postpartum OR "post-partum" OR pregnan* OR prenatal OR "pre-natal" OR primipara OR primiparous OR primigravida OR puerperium OR puerperal OR womb
24. 1 AND 2 AND 3
25. 1 AND 2 AND 4
26. **Ovid-Embase**
27. Angola OR Benin OR Botswana OR Burkina Faso OR Burundi OR Cameroon OR Cape Verde OR Central African Republic OR Chad OR Comoros OR Congo OR Cote d'Ivoire OR Ivory Coast OR Djibouti OR Equatorial Guinea OR Eritrea OR Ethiopia OR Gabon OR The Gambia OR Ghana OR Ghanian OR Guinea OR Guinea-Bissau OR Kenya OR Lesotho OR Liberia OR Madagascar OR Malawi OR Mali OR Mauritania OR Mauritius OR Mozambique OR Namibia OR Niger OR Nigeria OR Reunion OR Rwanda OR Senegal OR Seychelles OR Sierra Leone OR Somalia OR South Africa* OR Sudan OR Swaziland OR Tanzania OR Togo OR Uganda OR Western Sahara OR Zambia OR Zimbabwe
28. Albinism OR Albino OR muzungu OR oculocutaneous OR hypopigmention
29. Aunt OR anesthesiologist OR anaesthesiologist OR birth attendant OR Brother OR carer OR companion OR counsellor OR counselor OR Cousin OR Dermatologist OR doctor OR family OR families OR father OR grandmother OR gynecologist OR gynaecologist OR healer OR healthcare OR health care OR husband OR in-law OR Mama OR midwife OR midwives OR Mother OR neonatologist OR nurse OR obstetrician OR Ophthalmologist OR Paediatrician OR Pediatrician OR partner OR physician OR relative OR sibling OR sister OR social worker OR student OR TBA OR traditional OR woman OR women
30. Anesthesiologist OR anaesthesiologist OR birth attendant OR Brother OR carer OR companion OR counsellor OR counselor OR antepartum OR ante-partum OR antenatal OR ante-natal OR baby OR babies OR birth OR birthing OR caesarean OR cesarean OR c-section OR childbearing OR child-bearing OR Childbirth OR Deliver Or Delivery OR gestation OR infant OR intrapartum OR intra-partum OR labour OR maternal OR Maternity OR multigravida OR multi-gravida OR multipara OR multi-para OR multiparous OR multi-parous OR neonate OR neo-nate OR neonatal OR neo-natal OR newborn OR new-born OR obstetric OR perinatal OR peri-natal OR postnatal OR post-natal OR postpartum OR post-partum OR pregnancy OR pregnant OR prenatal OR pre-natal OR primipara OR primiparous OR primigravida OR puerperium OR puerperal OR womb
31. **PsychInfo**
32. Africa* OR "sub-Saharan" OR Angola OR Benin OR Botswana OR "Burkina Faso" OR Burundi OR Cameroon OR "Cape Verde" OR "Central African Republic" OR Chad OR Comoros OR Congo OR "Côte d'Ivoire" OR "Ivory Coast" OR Djibouti OR "Equatorial Guinea" OR Eritrea OR Ethiopia OR Gabon OR "The Gambia" OR Ghana OR Ghanian OR Guinea OR "Guinea-Bissau" OR Kenya OR Lesotho OR Liberia OR Madagascar OR Malawi* OR Mali OR Mauritania OR Mauritius OR Mozambique OR Namibia OR Niger OR Nigeria OR Réunion OR Rwanda OR "Sao Tome and Principe" OR Senegal OR Seychelles OR "Sierra Leone" OR Somalia OR "South Africa*" OR Sudan OR Swaziland OR Tanzania OR Togo OR Uganda OR "Western Sahara" OR Zambia OR Zimbabwe
33. DE "Albinism" OR Albinism OR Albino* OR achrom* OR muzungu OR oculocutaneous OR hypopigment*
34. DE "Adolescent Mothers" OR DE "Alternative Medicine" OR DE "Allied Health Personnel" OR DE "Anesthesiology" OR DE "Biological Family" OR DE "Brothers" OR DE "Caregivers" OR DE "Counselors" OR DE "Couples" OR DE "Cousins" OR DE "Expectant Mothers" OR DE "Family" OR DE "Family Members" OR DE "Family Physicians" OR DE "Fathers" OR DE "Father Child Relations" OR DE "Grandparents" OR DE "Gynecologists" OR DE "Health Personnel" OR DE "Human Females" OR DE "Husbands" OR DE "Institutional Attendants" OR DE "Medical Students" OR DE "Midwifery" OR DE "Mothers" OR DE "Mother Child Relations" OR DE "Nuclear Family" OR DE "Nurses" OR DE "Nursing" OR DE "Nursing Students" OR DE "Obstetricians" OR DE "Obstetrics" OR DE "Offspring" OR DE "Only Children" OR DE "Ophthalmology" OR DE "Optometrists" OR DE "Optometry" OR DE "Parent Child Relations" OR DE "Parent Training" OR DE "Parental Role" OR DE "Parenting" OR DE "Parents" OR DE "Parent Child Relations" OR DE "Pediatricians" OR DE "Partners" OR DE "Pediatricians" OR DE "Pediatrics" OR DE "Peers" OR DE "Physicians" OR DE "Psychiatric Social Workers" OR DE "Sexual Partners" OR DE "Siblings" OR DE "Single Mothers" OR DE "Sisters" OR DE "Social Support" OR DE "Social Workers" OR DE "Students" OR DE "Women's Rights" OR Aunt* OR anesthesiologist* OR anaesthesiologist* OR "birth attendant*" OR Brother* OR carer* OR companion* OR counsellor OR counselor OR Cousin OR Dermatologist OR docto* OR famil* OR father* OR grandmother* OR gynecologist OR gynaecologist OR healer* OR healthcare OR "health care" OR husband* OR "in-law*" OR Mama* OR midwi* OR Mother* OR neonatologist* OR nurs* OR obstetrician* OR Ophthalmologist OR Paediatrician OR Pediatrician OR partner* OR physician* OR relative* OR "RN" OR sibling* OR sister* OR "social work*" OR student* OR TBA OR tradition* OR woman OR women
35. DE "Adolescent Pregnancy" OR DE "Antepartum Period" OR DE "Birth" OR DE "Birth Trauma" OR DE "Caesarean Birth" OR DE "Child Welfare" OR DE "Childbirth Training" OR DE "Community Health" OR DE "Health Care Access" OR DE "Health Care Delivery" OR DE "Health Care Services" OR DE "Intrapartum Period" OR DE "Labor (Childbirth)" OR DE "Natural Childbirth" OR DE "Neonatal Intensive Care" OR DE "Neonatal Period" OR DE "Obstetrics" OR DE "Parents" OR DE "Perinatal Period" OR DE "Postnatal Period" OR DE "Pregnancy" OR DE "Pregnancy Outcomes" OR DE "Premature Birth" OR DE "Prenatal Care" OR DE "Primary Health Care" OR DE "Reproductive Health Care" OR DE "Reproductive Health Care" OR DE "Prenatal Care” OR DE "Primipara" OR DE "Private Sector" OR DE "Rural Health" OR DE "Urban Health" OR antepartum OR ante-partum OR antenatal OR "ante-natal" OR baby OR birth* OR caesarean OR cesarean OR "c-section" OR childbearing OR "child-bearing" OR Childbirth OR Deliver* OR gestation OR infant OR intrapartum OR "intra-partum" OR "labour and delivery" OR "labor and delivery" OR maternal OR Maternity OR multigravida OR "multi-gravida" OR multipara OR "multi-para" OR multiparous OR "multi-parous" OR neonate OR "neo-nate" OR neonatal OR "neo-natal" OR newborn OR "new-born" OR obstetric* OR perinatal OR "peri-natal" OR postnatal OR "post-natal" OR postpartum OR "post-partum" OR pregnan* OR prenatal OR "pre-natal" OR primipara OR primiparous OR primigravida OR puerperium OR puerperal OR womb
36. 1 AND 2 AND 3
37. 1 AND 2 AND 4
38. **Web of Science**
39. TS=(Africa* OR "sub-Saharan" OR Angola OR Benin OR Botswana OR "Burkina Faso" OR Burundi OR Cameroon OR "Cape Verde" OR "Central African Republic" OR Chad OR Comoros OR Congo OR "Côte d'Ivoire" OR "Ivory Coast" OR Djibouti OR "Equatorial Guinea" OR Eritrea OR Ethiopia OR Gabon OR "The Gambia" OR Ghana OR Ghanian OR Guinea OR "Guinea-Bissau" OR Kenya OR Lesotho OR Liberia OR Madagascar OR Malawi* OR Mali OR Mauritania OR Mauritius OR Mozambique OR Namibia OR Niger OR Nigeria OR Réunion OR Rwanda OR "Sao Tome and Principe" OR Senegal OR Seychelles OR "Sierra Leone" OR Somalia OR "South Africa*" OR Sudan OR Swaziland OR Tanzania OR Togo OR Uganda OR "Western Sahara" OR Zambia OR Zimbabwe)
40. TS=(Albinism OR Albino* OR achrom* OR muzungu OR oculocutaneous OR hypopigment*)
41. TS=(Aunt* OR Anesthesiologist* OR anaesthesiologist OR "birth attendant*" OR Brother* OR carer* OR companion* OR counsellor* OR counselor* OR Cousin* OR Dermatologist* OR docto* OR famil* OR father* OR grandmother* OR gynecologist* OR gynaecologist* OR healer* OR healthcare OR "health care" OR husband* OR "in-law*" OR Mama* OR midwi* OR Mother* OR neonatologist* OR nurs* OR obstetrician* OR Ophthalmologist* OR Paediatrician* OR Pediatrician* OR partner* OR physician* OR relative* OR sibling* OR sister* OR "social work*" OR student* OR TBA* OR tradition* OR woman OR women)
42. TS=(antepartum OR ante-partum OR antenatal OR "ante-natal" OR baby OR babies OR birth* OR caesarean OR cesarean OR "c-section" OR childbearing OR "child-bearing" OR Childbirth OR Deliver* OR gestation OR infant* OR intrapartum OR "intra-partum" OR "labour and delivery" OR "labor and delivery" OR maternal OR Maternity OR multigravida OR "multi-gravida" OR multipara OR "multi-para" OR multiparous OR "multi-parous" OR neonate OR "neo-nate" OR neonatal OR "neo-natal" OR newborn* OR "new-born*" OR obstetric* OR perinatal OR "peri-natal" OR postnatal OR "post-natal" OR postpartum OR "post-partum" OR pregnan* OR prenatal OR "pre-natal" OR primipara OR primiparous OR primigravida OR puerperium OR puerperal OR womb)
43. 1 AND 2 AND 3
44. 1 AND 2 AND 4
45. **Global Index Medicus**
46. Albinism OR Albino* OR achrom* OR muzungu OR oculocutaneous OR hypopigment*
