# Supplementary material for: A participatory systematic review on human rights and the birth of a child with albinism in sub-Saharan Africa
Source: Womens Health (Lond). 2025 Dec 11;21:17455057251395420. doi: 10.1177/17455057251395420 (PMC12699010; doi:10.1177/17455057251395420)
Supplement: sj-docx-2-whe-10.1177_17455057251395420 – Supplemental material for A participatory systematic review on human rights and the birth of a child with albinism in sub-Saharan Africa [file sj-docx-2-whe-10.1177_17455057251395420.docx]

**Supplemental File: Included Articles**

| **Citation and Article Type** | **Discipline or field of study of author(s)** | **Country of author(s) and Geographical Focus** | **Focus of article/ Research purpose/ Research question** | **Birthing-specific (perinatal-focused) Evidence** | **Quality Appraisal* and**  **Level of Evidence**** |
| --- | --- | --- | --- | --- | --- |
| Aquaron R, Djatou M and Kamdem L. Socio-cultural aspects of albinos in Black Africa: Mutilations and ritual crimes perpetrated in East Africa (Burundi and Tanzania). *Medecine Tropicale: Eevue du Corps de Sante Colonial* 2009; 69: 449-453.  Article type: Discussion | Molecular Biology, Human Sciences, Medicine | Country of author(s):  France  Geographical region of focus:  Sub-Saharan Africa | “This report presents an overview of the many socio-cultural prejudices confronting albinos and their parents in Sub-Saharan Africa at each stage of life (infancy, adolescence, and adulthood)”. (p. 449) | A substantive one paragraph describing the causation of the birth of a child with albinism with layers of discussion on beliefs about the condition; discussed in locations not often mentioned in the literature: Cameroon, Congo | Quality Appraisal: 6/6  Level of evidence: 5 |
| Baker C, Lund P, Nyathi R, et al. The myths surrounding people with albinism in South Africa and Zimbabwe. *Journal of African Cultural Studies* 2010; 22: 169-181. DOI: 10.1080/13696815.2010.491412.  Article Type: Qualitative | European Languages and Cultures; Biomolecular and Sports Science; Advocacy; Nursing/ Midwifery | Country of author(s):  UK & Zimbabwe  Geographical region of focus:  South Africa, Zimbabwe | "This article has demonstrated that the attitudes of friends, family, colleagues and healthcare professionals, whether positive or negative, can signiﬁcantly inﬂuence the life experience of people with albinism. Therefore, the importance of community responsibility for challenging the myths that surround albinism, and the provision of the necessary support for people with albinism to be able to manage their condition effectively cannot be underestimated." (p. 178) | 2-page (substantive) section on birth of a baby with albinism includes discussion of positive experience of genetic counselling in a large urban hospital; rural setting more likely to experience myths around causation | Quality Appraisal: 9/10  Level of evidence: 3 |
| Benyah F. Equally able, differently looking  discrimination and physical violence against persons with albinism in Ghana. *Journal for the Study of Religion* 2017; 30: 161-188.  Article type: Discussion | Religious Studies | Country of author(s):  Ghana  Geographical region of focus:  Ghana | "The question is: how do religious beliefs and cultural values contribute to the plights of albinos in Ghana? What is the place of albinos within Ghanaian socio-religious space? And, how can the inculturation of human rights help mitigate the violence against albinos in Ghana?" (p. 165). | Seven sentences describing the myths on the birth of a baby with albinism. | Quality Appraisal: 6/6  Level of evidence: 5 |
| Braathen SH and Ingstad B. Albinism in Malawi: Knowledge and beliefs from an African setting. *Disability & Society* 2006; 21: 599-611. DOI: 10.1080/09687590600918081.  Article Type: Qualitative | Health Research, Living Conditions, and Service Delivery | Country of author(s):  Norway  Geographical region of focus:  Malawi | “…to examine knowledge, beliefs and behaviour related to people living with albinism in Malawi” (p. 599) | Four sentences that spoke to the myths about a birth of a child with albinism—mainly to describe the beliefs about albinism | Quality Appraisal: 7/10  Level of evidence: 3 |
| Bradbury-Jones C, Ogik P, Betts J, et al. Beliefs about people with albinism in Uganda: A qualitative study using the Common-Sense Model. *PloS One* 2018; 13: e0205774. DOI: 10.1371/journal.pone.0205774.  Article Type: Qualitative | Nursing, Advocacy,  Life Sciences | Country of author(s):  United Kingdom, Uganda  Geographical region of focus:  Uganda | This article reports on the findings of a qualitative study conducted in Uganda that addressed the following question: What are the beliefs that surround people with albinism in Uganda? | A substantiative section in their findings shared the causation of albinism with ghost or mother blaming narrative. This article is one of the only articles that shared first-hand account of a father upon the birth of his child with albinism. | Quality Appraisal: 10/10  Level of evidence: 3 |
| Brocco G. Labeling albinism: Language and discourse surrounding people with albinism in Tanzania. *Disability & Society* 2015; 30: 1143-1157. DOI: 10.1080/09687599.2015.1075869.  Article Type: Qualitative | Medical Anthropology | Country of author(s):  Germany  Geographical region of focus:  Tanzania | "The article will show how persons with albinism try to explain and conceptualize their own physical condition by identifying themselves as disabled and by adopting institutional terminology generated by western media campaigns and debates on their behalf." (p.1144) | This article primarily provides a-page description surrounding a birth of a baby with albinism: mother blaming and that if there is a relative with the condition, it leads to acceptance. Mothers also describe this as a plan of God. | Quality Appraisal: 8/10  Level of evidence: 3 |
| Brocco G. Albinism, stigma, subjectivity and global-local discourses in Tanzania. *Anthropology & Medicine* 2016; 23: 229-243. DOI: 10.1080/13648470.2016.1184009.  Article Type: Qualitative | Medical Anthropology | Country of author(s):  Germany  Geographical region of focus:  Tanzania | "The analysis endeavors to show how subjectivity is inﬂuenced by the experiences of everyday interactions with other people (mainly characterized by visible and invisible stigmatizing or accepting behaviors) and imagined horizons. In so doing, the research sheds light on the self-perceptions of people with albinism regarding the condition and how this affects their daily interactions with others, as well as the societal attitudes toward them." (p.v231) | Gives a page to share causation of albinism—mostly accounts from persons with albinism (recounting what their parents’ experiences were when they were born). | Quality Appraisal: 8/10  Level of evidence: 3 |
| Brocco G. Assemblages of care around albinism  Kin-based networks and (in)dependence in contemporary Tanzania. In: Montesi L and Calestani M (eds) *Managing Chronicity in Unequal States*. UCL Press, 2021, pp.112-131.  Article type: Discussion | Medical Anthropology | Country of author(s):  Germany  Geographical region of focus:  Tanzania | “In light of the killings and the subsequent media/humanitarian publicity, the present analysis has a double goal. First, it sheds light on how my interlocutors with albinism navigated and interacted with various forms of care and dependence, enacted within and shaped by their kin-based networks, neighbours, religious congregations, national and international organisations, and the Tanzanian nation state. The second goal is to reveal how notions of care over the last decades have shifted from being characterised as communitarian practices to individualised endeavours enmeshed within narratives of national development (*maendeleo*) and a spirit of (individual) empowerment (*kuwezesha*).” (p. 113) | A page and a half recounting one positive experience at home and in their community upon the birth of a baby with albinism attributed to God’s will and known lineage of albinism in family but also acknowledging known beliefs on causations like curse or mother’s past wrongdoing. | Quality Appraisal: 6/6  Level of evidence: 5 |
| Bryceson DF, JØNsson JB and Sherrington R. Miners 'magic: Artisanal mining, the albino fetish and murder in Tanzania. *Journal of Modern African Studies* 2010; 48: 353-382. DOI: 10.1017/S0022278X10000303.  Article type: Discussion | Sociologist/  geographer, anthropologist | Country of author(s):  Scotland, Denmark, United Kingdom  Geographical region of focus:  Tanzania | “Through the concept of fetish creation, this article interrogates the agency of those involved in the murders: the miners who purchase the albino charms, the *waganga* healers renowned for their healing, divination and sorcery skills who prescribe and sell the charms, and the albino murder victims. The agrarian background, miners’ ambitions and a clash of values comprise our starting point for understanding the victimisation of albinos.” (p. 354) | Two paragraphs on reported practices in killing persons with albinism at birth and the tension that arises in the family upon the birth. | Quality Appraisal: 5/6  Level of evidence: 5 |
| Cruz-Inigo AE, Ladizinski B and Sethi A. Albinism in Africa: Stigma, slaughter and awareness campaigns. *Dermatologic Clinics* 2011; 29: 79-87. DOI: 10.1016/j.det.2010.08.015  Article type: Discussion | Dermatology, Internal Medicine | Country of author(s):  USA  Geographical region of focus:  Sub-Saharan Africa | “It is imperative to inform the medical community and the general national and international public about the tragedies faced by albinos to protect them from skin cancer and ritualistic murders by individuals seeking wealth through clandestine markets perpetuating witchcraft.” (p. 79) | A sentence reporting on midwives killing persons with albinism at birth and enumeration of causations of their birth. Then a paragraph on birth involves proposed solutions on health education and genetic care programs. | Quality Appraisal: 6/6  Level of evidence: 5 |
| de Pina-Cabral J. Albinos do not die: Belief, philosophy and anthropology. In: Giri AK and Clammer J (eds) *Philosophy and Anthropology*. Anthem Press, 2013, pp.305-322.  Article type: Discussion | Social Anthropology | Country of author(s):  Portugal  Geographical region of focus:  Mozambique | “In Mozambique one is often told things about albinos that can hardly be interpreted at face value...Upon hearing them, I was immediately challenged by the following question: if these beliefs do not meet up with the test of disbelief, what then is the significance of both conveying and holding them?” (p. 305) | Two paragraphs about men leaving their wives after birth due to different colour of skin and hair as well a story of a mother who killed two of her babies with albinism at birth. | Quality Appraisal: 6/6  Level of evidence: 5 |
| Ibhawoh B, Reimer-Kirkham S, Ero I, et al. Shifting wrongs to rights: Lessons in human rights from the situation of mothers impacted by slbinism in Africa. *Journal of Human Rights Practice* 2022; 14: 838-858. DOI: 10.1093/jhuman/huac038.  Article Type: Qualitative | Human rights, law, nursing | Country of author(s):  Tanzania Canada, Nigeria, UK  Geographical region of focus:  Tanzania and South Africa | "In this paper, scholars and advocates from the Global South and Global North analyse the challenges in the practice of human rights, with a focus on mothers impacted by albinism." p. 3 | Seven sentences touch on an experience of a pastor and his faith community providing support to a mother who had a baby with albinism and a mother reported she was denied disability grant because her baby was not recognized to have a disability. | Quality Appraisal: 10/10  Level of evidence: 3 |
| Imafidon E. Dealing with the other between the ethical and the moral: Albinism on the African continent. *Theoretical Medicine and Bioethics* 2017; 38: 163-177. DOI: 10.1007/s11017-017-9403-2.  Article type: Discussion | African Philosophy | Country of author(s):  United Kingdom/  Nigeria  Geographical region of focus:  Africa | “My primary interest in this article is to explore how the uneasiness and the consequent indifference about the other play out on the African continent with particular attention to albinism. I am particularly interested in showing how the African community has managed to establish and promote ontological and normative ideologies that help sustain the (ill) treatment of persons with albinism as an other.” (p. 164) | Four sentences describe that albinism is seen as ghosts or dead persons returned as while babies. This article however provides context on African ontology relating to when a child is born with deformities and seen as not human beings. | Quality Appraisal: 6/6  Level of evidence: 5 |
| Kayombo EJ. Belief on witchcraft and killing of people with albinism in Lack Victoria Zone, Tanzania. *Journal of Scientific Research and Reports* 2021; 27: 33-46.  Article Type: Qualitative | Traditional Medicine | Country of author(s):  Tanzania  Geographical region of focus:  Tanzania | “The aim of this paper is to explore other factors that contribute to killing old people and people with albinism. The main focus were to explore factors that lead to killing old people and people with albinism in addition to current belief of witchcraft.” (p. 33) | The findings include a substantiative section (3 paragraphs) of experiences of a mother giving birth to a baby with albinism: abandonment, killings, and selling of PWA. | Quality Appraisal: 9/10  Level of evidence: 3 |
| Kiluwa SH, Yohani S and Likindikoki S. Accumulated social vulnerability and experiences of psycho-trauma among women living with albinism in Tanzania. *Disability & Society* 2022: 1-22. DOI: 10.1080/09687599.2022.2072706.  Article Type: Qualitative | Psychology and Counseling, Educational Psychology, Psychiatry and Mental Health | Country of author(s):  Tanzania, Canada  Geographical region of focus:  Tanzania | “Based on the limited psychosocial research on people with albinism in Tanzania, this exploratory study aimed to deepen our understanding of the experiences of women with albinism residing in Dar es Salaam, Tanzania, with a focus on their challenges. This study also contributes to research on people with albinism in Tanzania by examining the unique experiences of women from their voices.” (p. 4) | Seven sentences about a child killed at birth and an experience of a person with albinism whose parents rejected her at birth. | Quality Appraisal: 8/10  Level of evidence: 3 |
| Kromberg JGR and Kerr R. Oculocutaneous albinism in southern Africa: Historical background, genetic, clinical and psychosocial issues. *African Journal of Disability* 2022; 11: 1-7. DOI: 10.4102/ajod.v11i0.877.  Article type: Discussion | Human Genetics | Country of author(s):  South Africa  Geographical region of focus:  Southern Africa | “This article provides a historical background on oculocutaneous albinism (OCA) in southern Africa and presents relevant information from the literature regarding epidemiology, genetics and genetic counselling, health, psychosocial and cultural issues, and medical care.” (p. 1) | One paragraph on the shock and upset emotions mothers feel, referencing authors’ earlier work (Kromberg et al., 1987) and a paragraph on recommendation that directly relates to the first year of life of a baby with albinism. | Quality Appraisal: 6/6  Level of evidence: 5 |
| Kromberg JGR, Manga P and Kerr R. Children with oculocutaneous albinism in Africa: Characteristics, challenges and medical care. *South African Journal of Child Health* 2020; 14: 50-54. DOI: 10.7196/SAJCH.2020.v14.i1.1608.  Article type: Discussion | Human Genetics, Molecular Biology | Country of author(s):  (1) South Africa (2) South Africa/USA (3) South Africa  Geographical region of focus:  Southern Africa | “This review aims to explore the latest literature available on the epidemiology, genetics, clinical characteristics, psychosocial issues and possible management strategies, focusing on affected children.” (p. 50) | Three paragraphs on birth describing the ignorance and misinformation about albinism and limited knowledge of the genetics on albinism. | Quality Appraisal: 6/6  Level of evidence: 5 |
| Kromberg JG, Zwane EM and Jenkins T. The response of black mothers to the birth of an albino infant. *American Journal of Diseases of Children* 1987; 141: 911-916. DOI: 10.1001/archpedi.1987.04460080097038.  Article Type: Qualitative | Human Genetics, Nursing | Country of author(s):  South Africa  Geographical region of focus:  Southern Africa | “The aim of the present study was to investigate prospectively the response of a black mother to her newborn albino infant and to follow and study the dyad longitudinally until the infant reached 15 months of age.” (p. 911) | This study’s main focus on mothers’ experiences of having a baby with albinism in South Africa. | Quality Appraisal: 10/10  Level of evidence: 3 |
| Likumbo N, de Villiers T and Kyriacos U. Malawian mothers' experiences of raising children living with albinism: A qualitative descriptive study. *African Journal of Disability* 2021; 10: 693. DOI: 10.4102/ajod.v10i0.693.  Article Type: Qualitative | Nursing & Midwifery | Country of author(s):  South Africa  Geographical region of focus:  Malawi | “This study aims to describe Malawian mothers’ experiences, perceptions and understanding of raising children with albinism (CWA).” (p. 1) | The findings of the study predominantly shared mothers’ experiences during birth and the first few years of the child with albinism. | Quality Appraisal: 9/10  Level of evidence: 3 |
| Lund PM. Oculocutaneous albinism in southern Africa: population structure, health and genetic care. *Annals of Human Biology* 2005; 32: 168-173. DOI: 10.1080/03014460500075423.  Article type: Discussion | Biosciences, Genetics | Country of author(s):  England/  South Africa  Geographical region of focus:  South Africa and Zimbabwe | “This review reports population studies in southern Africa to determine the distribution of people with OCA in this predominantly rural and relatively impoverished area—crucial information for health and education providers.” (p. 168) | One paragraph explaining the genetic aspects of albinism and the need for genetic outreach programs to train health professionals in diagnosing albinism and provide education to the mother about albinism. | Quality Appraisal: 6/6  Level of evidence: 5 |
| Lynch P, Lund P and Massah B. Identifying strategies to enhance the educational inclusion of visually impaired children with albinism in Malawi. *International Journal of Educational Development* 2014; 39: 216-224.  Article Type: Qualitative | Education, Bioscience, Advocacy | Country of author(s):  (1) United Kingdom, (2) United Kin  gdom/South Africa (3) Malawi  Geographical region of focus:  Malawi | “In order to help address the paucity of data on the lives of people with albinism, a small team of Malawian and UK researchers carried out a study to collect primary data about school children’s experiences living with the condition in different educational settings in Malawi.” (p. 226) | Cumulatively, it has a page that give insights to a birth of a child with albinism including first-hand accounts of mothers’ and family’s experiences. Notably, this article share positive responses about the child such that it is God’s will. | Quality Appraisal: 7/10  Level of evidence: 3 |
| Machoko CG. Albinism: A life of ambiguity – a Zimbabwean experience. *African Identities* 2013; 11: 318-333. DOI: 10.1080/14725843.2013.838896.  Article Type: Qualitative | Religious Studies | Country of author(s):  Canada  Geographical region of focus:  Zimbabwe | “I argue that people with albinism lived in ambiguity. I also assert that the double meaning happened because albinism was linked to water spirits and ascribed/ notional celibacy. I also maintain that the biggest obstacle preventing people with albinism from taking full part in Zimbabwean society derives from African traditional religious myths and beliefs, which made them to live in ambiguity.” (p. 318) | In total, a page accounted for experiences of a birth of a child with albinism, with the emphasis on African cosmology and stories on infanticide. | Quality Appraisal: 77/10  Level of evidence: 3 |
| Mostert MP. The plight of Africans with albinism. *Human Life Review* 2019; 45: 56-62.  Article type: Discussion | Education, Disability, Bioethics | Country of author(s):  United States  Geographical region of focus:  Africa | “While much of the developed world has made significant progress in including people with medical and physical disabilities, there are many places where this is not the case. The continued ostracism and abuse of Africans with albinism requires urgent action so that these people may take their rightful place as valued members of their communities and societies.” (p. 61) | Five sentences that talked broadly about the killings of a baby with albinism and the blame towards the mother. | Quality Appraisal: 6/6  Level of evidence: 5 |
| Munyere A. Living with a disability that others do not understand. *British Journal of Special Education* 2004; 31: 31-32. DOI: 10.1111/j.0952-3383.2004.00323.x.  Article type: Discussion | Inclusive Education | Country of author(s):  Kenya  Geographical region of focus:  Kenya | “In this article, Alex Munyere recalls his childhood and his life at school. He provides a fascinating first-hand account of the experience of living with a disability.” (p. 31) | Two paragraphs describing the response of a father and community to the birth of a child with albinism, with an emphasis on traditional practices. | Quality Appraisal: 4/6  Level of evidence: 5 |
| Ngula A. The power of information and coping with albinism: An autoethnographic study. *IFLA Journal* 2023; 49: 432-442.  Article Type: Qualitative | Library Sciences | Country of author(s):  Namibia  Geographical region of focus:  Namibia | “This article aims to ascertain the purposes for which the author needed information when her children with albinism were born, as well as explore the mechanisms she used to find information and the challenges she experienced when searching for information.” (p. 1) | Five paragraphs in which the author, a person with albinism, shared the experiences of his family when he was born and traditions during a time of birth like that a father can show his disapproval of the child if he does not believe it to be his. | Quality Appraisal: 9/10  Level of evidence: 3 |
| Nyamu IK. Competing intergenerational perspectives of living with albinism in Kenya and their implications for children’s lives. *Childhood* 2020; 27: 435-449.  Article Type: Qualitative | Inclusive Education | Country of author(s):  South Africa  Geographical region of focus:  Kenya | “Using generationing as a theoretical lens, this article explores how generational relationships mediate children’s experiences of living with albinism in the context of harmful cultural practices, disability politics and adult-defined activism.” (p. 435) | One substantial paragraph with reports of a mother who killed her baby, and reports of partner and family abandonment. | Quality Appraisal: 10/10  Level of evidence: 3 |
| Ojilere A and Saleh MM. Violation of dignity and life: Challenges and prospects for women and girls with albinism in sub-Saharan Africa. *Journal of Human Rights and Social Work* 2019; 4: 147-155.  Article type: Discussion | Law | Country of author(s):  Nigeria  Geographical region of focus:  Sub-Saharan Africa | “Using a doctrinal methodology and drawing instances from across Sub-Saharan Africa, this paper supports recent findings that persecution, social marginalization, and violence sum the fate of women with albinism in Africa.” (p. 148) | Six sentences that talked broadly about the birth of a child with albinism and are killed for ritual purposes. While this paper centres on women with albinism, their findings and discussion point towards public health programs to address the reactions at birth. | Quality Appraisal: 6/6  Level of evidence: 5 |
| Reimer-Kirkham S, Astle B, Ero I, et al. Mothering, albinism and human rights: The disproportionate impact of health-related stigma in Tanzania. *Foundations of Science* 2020; 27: 719–740.  Article Type: Qualitative | Nursing, (3) Human rights and law, Philosophy | Country of author(s):  (1,2,5) Canada, (3,4) Nigeria  Geographical region of focus:  Tanzania | "In this paper we present an analysis of mothers’ experiences of stigma, drawing on a critical ethnography on mothering, albinism, and human rights that is underway in Tanzania" (p. 2) | Approximately three pages reported first-hand accounts of mothers’ birthing experiences, including reactions from partners, health providers and community. | Quality Appraisal: 10/10  Level of evidence: 3 |
| Reimer-Kirkham S, Astle B, Ero I, et al. Albinism, spiritual and cultural practices, and implications for health, healthcare, and human rights: A scoping review. *Disability & Society* 2019; 34: 747-774. DOI: 10.1080/09687599.2019.1566051.  Article type: Scoping Review | Nursing, Human rights and law, Philosophy | Country of author(s):  (1,2,4,5) Canada (3) Nigeria  Geographical region of focus:  Worldwide, primarily Africa | "To support this mandate of the UN Independent Expert on the Enjoyment of Human Rights by Persons with Albinism, we undertook a scoping review to map the field of research on albinism, spiritual and cultural practices, and healthcare. In this article, we provide a short background on the genetic condition of albinism, describe the scoping review method, and summarize our findings." (p. 2) | Two paragraphs that reported on the causation of albinism relating to birth such as mothers’ fault or a punishment. | Quality Appraisal: 9/10  Level of evidence: 2 |
| Reimer-Kirkham S, Astle B, Kromberg J, et al. Birth stories of South African mothers of children with albinism: A critical human rights analysis. *International Journal of Africa Nursing Sciences* 2024; 20: 100650.  Article Type: Qualitative | Nursing, advocates, Human rights | Country of author(s):  Canada & South Africa  Geographical region of focus:  South Africa | "In this paper, we present the findings of a study exploring the resilience of mothers in South Africa impacted by albinism. We do so through a human rights lens, focusing on the perinatal period and building on the earlier ground-breaking work of Dr. Jennifer Kromberg." (p.1) | This entire article focuses on the birth of a baby with albinism in South Africa. | Quality Appraisal: 10/10  Level of evidence: 3 |
| Reimer-Kirkham S, Ero I, Astle B, et al. UN resolution on the elimination of harmful practices and the protection of human rights of mothers impacted by albinism. *Journal of Global Health* 2022; 12. DOI: 10.7189/jogh.12.03029.  Article type: Discussion | Nursing, Human rights and law, Philosophy | Country of author(s):  Canada, Nigeria  Geographical region of focus:  Worldwide | The article focuses on the relevance of the UN resolution on the Elimination of Harmful Practices related to Accusations of Witchcraft and Ritual Attacks to the experiences of PWA, particularly mothers impacted by albinism. | Discussion includes 2 sentences about the birthing experience of mothers. This brief commentary however provides guidance to how to address the accusations that mothers experience on having a child with albinism. | Quality Appraisal: 6/6  Level of evidence: 5 |
| Reimer-Kirkham S, Ero I, Mgijima-Konopi I, et al. Mothering and albinism: Recommendations for disability rights in Africa. *African Disability Rights Yearbook* 2021; 9: 283-292. DOI: 10.29053/2413-7138/2021/v9a14.  Article type: Discussion | Nursing, Human rights and law, Philosophy | Country of author(s):  Canada, Nigeria, South Africa  Geographical region of focus:  Africa | "This commentary focuses on the little researched experiences of mothers impacted by albinism, whether with albinism themselves or with children with albinism." (p. 284) | Four sentences that directly mention a birth of a child with albinism and mothers being blamed or carrying the burden resulting in gender-based violence and abandonment. This article, however, gives broader context to the trajectory of life for the mothers following the birth of their child with albinism. | Quality Appraisal: 6/6  Level of evidence: 5 |
| Tambala-Kaliati T, Adomako EB and Frimpong-Manso K. Living with albinism in an African community: Exploring the challenges of persons with albinism in Lilongwe District, Malawi. *Heliyon* 2021; 7: e07034. DOI: 10.1016/j.heliyon.2021.e07034.  Article Type: Qualitative | Social Work | Country of author(s):  Ghana  Geographical region of focus:  Malawi | “This study aimed to fill a research void by examining the problems faced by people with albinism in Malawi's Lilongwe District.” (p. 2) | Approximately a page (four paragraph) discussed the birth of a child with albinism, specifically in their findings. They discussed the misunderstanding around albinism and the lack of knowledge of mothers, families and communities. | Quality Appraisal: 8/10  Level of evidence: 3 |
| Taylor J, Bradbury-Jones C, Ogik P, et al. Reactions to and explanations for the birth of a baby with albinism: A qualitative study in Busoga, Uganda. *BMJ Open* 2021; 11: e040992. DOI: 10.1136/bmjopen-2020-040992.  Article Type: Qualitative | Nursing,  Social science | Country of author(s):  UK, Uganda  Geographical region of focus:  Uganda | "The analysis here is derived from a larger study across both Uganda and Tanzania that explored the impact of stigma and fear on education and life opportunities for people with albinism. We interrogated the Ugandan interview data to answer the following questions relating to those in attendance at the birth of a baby with albinism or, for those with albinism, who had been told the stories by their parents: 1. What were their birth stories, including reactions to the birth? 2. How was the birth of a child with albinism explained?" p.2 | This entire article focuses on the birth of a baby with albinism in Uganda. | Quality Appraisal: 10/10  Level of evidence: 3 |
| Taylor J, Bradbury‐Jones C and Lund P. Witchcraft‐related abuse and murder of children with albinism in sub‐Saharan Africa: A conceptual review. *Child Abuse Review* 2019; 28: 13-26. DOI: 10.1002/car.2549.  Article type: Discussion | Nursing, clinical science, social science | Country of author(s):  UK  Geographical region of focus:  Sub-Saharan Africa | “This review proposes a conceptual model to act as a platform for research on which to build and enhance our understanding of the lives of children with albinism in sub-Saharan Africa.” (p. 13) | Nine sentences that directly mentions the birth of a child with albinism including causation and reported killings of babies with albinism. |  |
| * Using Joanna Briggs Institute’s [Critical Appraisal Tools](https://jbi.global/critical-appraisal-tools), corresponding to the type of source  ** Using Joanna Briggs Institute’s [Levels of Evidence for Meaningfulness](https://jbi.global/sites/default/files/2019-05/JBI-Levels-of-evidence_2014_0.pdf):  1. Quantitative or mixed-methods systematic review  2. Qualitative or mixed-methods synthesis  3. Single qualitative study  4. Systematic review of expert opinion  5. Expert opinion  See also Supplemental File Critical Appraisials for details. | | | | | |

**Grey Literature**

| **Reference** | **Organization (govt/ NGO/**  **author)** | **Discipline or field of study of author(s)** | **Country of author(s), if applicable** | **Region of focus** | **Focus of source/ Research purpose/ Research question** |
| --- | --- | --- | --- | --- | --- |
| Ackley C. *The fetishization of albinos in Tanzania.* University of Chicago, Chicago, 2010. | Author | Unknown | USA | Tanzania | "By focusing on the fetish of the albino, I illustrate the significance of whiteness, the efficacy of body parts, and the function of fishing (and, nets) in this moment of Tanzania’s post-colonial history. I propose a correlation between the fetishization of the white skin of albinos and that of the successful and wealthy Europeans involved in the fishing industry around Lake Victoria." (p. 9) |
| Africa Albinism Network. *Submission in response to call for input by the OHCHR on Social development challenges faced by persons with albinism, including the impact of the COVID-19 pandemic,* https://africaalbinismnetwork.org/wp-content/uploads/2022/09/163906177888998oqwk9pgyn-1.pdf (2021, accessed December 16 2024). | NGOs | n/a | Organizations are from Africa | “The submission will provide information on the situation in 10 African countries: namely Nigeria, Uganda, Rwanda, South Africa, Zimbabwe, Zambia, Mozambique, Ghana, Malawi and Benin. (p. 1) | This report is in response to call for input by the OHCHR on Social development challenges faced by persons with albinism, including the impact of the COVID-19 pandemic on access to health and social services and education. |
| Albinism Foundation of E.A. *Plight of persons living with albinism in Kenya,* https://africaalbinismnetwork.org/wp-content/uploads/2022/09/15326228284469zd5fev73qjpdq4j4lncj714i-1.pdf (n.d., accessed December 16 2024). | NGOs | n/a | Kenya | Kenya | The report provides information about attacks on persons with albinism and the stigma, discrimination, and exclusion faced on Kenya. |
| Allen K. *Oppression through omission: The human rights case of persons with albinism in Uganda,* http://www.underthesamesun.com/sites/default/files/Kelly%20Allen%27s%20research  %20on%20PWA%20in%20Uganda.pdf (2010, accessed December 16 2024). | Author | Development Studies | USA | Uganda | "This paper looks into what the specific and unique challenges are facing persons with albinism, particularly in Uganda, and how current Human Rights documents do and do not address those issues. The paper also explores the possible reasons why the issues surrounding albinism are only recently being discussed." (p. 1) |
| Amnesty International. *"We are not animals to be hunted or sold": Violence and discrimination against people with albinism in Malawi,* https://www.amnesty.org/en/documents/afr36/4126/2016/en/ (2018, accessed December 16 2024). | International organization | n/a | n/a | Malawi | "This report focuses on the lived experiences of people with albinism in Malawi in the context of superstition-driven attacks against them and the corresponding government failure to protect the right to life for this vulnerable group and to guarantee their right to security of person." (p.5) |
| Braathen SH. *Albinism in Malawi: A qualitative study on attitudes and beliefs*. Master’s thesis, University of Oslo, 2005. | Thesis | International Community Health, Medicine | Norway | Malawi | "Albinism in Malawi is a qualitative project that has set out to examine attitudes and beliefs related to people living with albinism in Malawi. The aim has been to assess what implications albinism has on the lives of those born with it, and for their closest network of family and friends." (p. 4). |
| Buyco M, Reimer-Kirkham S, Astle B, et al. Access to healthcare by mothers impacted by albinism in South Africa. *Southern Africa Albinism Journal*. in press. | authors | (1,2,3, 8, 10) nursing, (4) policy, (5) genetics, (8, 9) human rights, (7, 11, 12) NGOs | (1,2,3, 10) Canada & (4,5,6,7,8,9,11, 12) South Africa | South Africa | "In this paper, we focus on access to healthcare by mothers impacted by albinism in South Africa, and the inequalities and discrimination experienced by them. As a research-advocacy-policy network with team members from six countries, we are conducting ethnographic research on mothering and human rights, including mothers who themselves have albinism or have children with albinism." (p. 1). |
| Daklo AK. *Access to healthcare for persons with albinism in Ghana: A human rights approach*. Master's thesis, Global Campus of Human Rights, 2021. | Thesis | Human Rights | Ghana | Ghana | “This study aims to examine: I. The healthcare needs of persons with albinism in Ghana; II. The legal and institutional framework on the rights to access to health by persons with albinism in Ghana and their conformity to international human rights Law; III. The measures to be taken to protect, promote and fulfil the rights to health by persons with albinism under international hu-man rights law and best practices.” (p. 6) |
| Diale MS. *Caring for a child with albinism: A pastoral challenge*. Master's thesis, University of Pretoria, South Africa, 2021. | Thesis | Practical theology | South Africa | Africa | “The aim to:1.Investigate the misguided beliefs and sub-cultural myths around the birth, nurturing, and caring of children with Albinism. 2.To further consider what the pastoral care practitioners can offer to support them and their families. [The objectives was] To help create a link between communities, pastoral care practitioners, and families have children with Albinism, and provide a holistic approach to handling the pressure they endure in their lives.” (p. 6) |
| Ero I. *Persons with albinism in particular women. Women's rights in Africa,* https://africaalbinismnetwork.org/wp-content/uploads/2022/09/15341976333908pu6paqdc0ctr5gq9wyo6layvi-1.pdf (n.d., accessed December 16 2024). | author | n/a | n/a | Africa | The short discussion gives insight to persons with albinism, particularly the vulnerability of women impacted by albinism through intersectionality and disability lens. |
| Ero I, Muscati S, Boulanger A., et al. *People with albinism worldwide: A human rights perspective*, https://africaalbinismnetwork.org/wp-content/uploads/2022/09/16233308202568dmrg1twthx-1.pdf (2021, accessed December 16 2024). | organization | (1,2) human rights activist, law (3) policy analyst | Canada | Worldwide | "This report aims to provide a comprehensive account of the human rights situation of people with albinism around the world. A global account of the human rights situation of people with albinism will help to build solidarity among the global albinism community by illustrating the experiences that unite them. It will also bring visibility to human rights abuses, and thus support albinism communities, particularly those that face extreme human rights violations. The expectation is that this report will prompt much-needed research, data collection, and discussion on issues affecting people with albinism so that progress may be made in the respect, protection, and fulfilment of their human rights." (p.3) |
| Estes P. *Unexpected destiny: A story of albinism, adoption, cross-cultural living, and a search for identity*. WestBow Press, 2014. | Book | Unknown | USA | Africa | This book shares the story of Pat and her family, focusing on the adoption and life of her daughter Suzanne. She was adopted in Africa and the book shares her journey and coming back to her birth-family, their unfamiliar culture, and the African village of her roots. |
| Human Rights Office of the High Commissioner of Human Rights. *Enhancing equality and countering discrimination against persons with albinism in Uganda,* https://africaalbinismnetwork.org/wp-content/uploads/2022/09/1571239746249c57w1jwdnh8-1.pdf (2019, accessed December 16 2024). | International organization | n/a | n/a | Uganda | "The study objective is to identify credible data that will enable a better understanding and appreciation of albinism issues and provide a credible source of information regarding the status of persons with albinism in Uganda, and to inform policy, planning and design of effective strategies for addressing challenges that affect the albinism community in the country." (p. 6-7) |
| International Bar Association. "*Waiting to disappear": International and regional standards for the protection and promotion of the human rights of persons with albinism,* https://africaalbinismnetwork.org/wp-content/uploads/2022/09/1535142781025ecwo3w6qfjn2kqgd5web9ms4i-1.pdf (2017, accessed December 16 2024). | organization | Human rights/policy | From their website, they are a "global legal community." | worldwide | "The paper provides a thorough overview of applicable international and regional laws, norms and interpretative frameworks with the goal of providing technical assistance for legal practitioners, human rights bodies and mechanisms." (p. 8) |
| International Federation of Red Cross and Red Crescent Societies. *Through albino eyes: The plight of albino people in Africa’s Great Lakes region and a Red Cross response –advocacy report,* https://www.ifrc.org/Global/Publications/general/177800-Albinos-Report-EN.pdf (2009, accessed December 16 2024). | International organization | n/a | n/a | Tanzania | "Yet this report, based on detailed field research in Tanzania and Burundi, shows that many ordinary villagers in remote areas have done their best to protect people among them who live with albinism and who are also their husbands, wives, parents or, most often, their children." (p. 3) |
| Kromberg JGR. Introduction and historical background. In: Kromberg JGR and Manga P (eds) *Albinism in Africa: Historical, geographic, medical, genetic,* *and psychosocial aspects*. Academic Press, 2018. | Book chapter | Human Genetics | South Africa | Africa | “This chapter will outline the relevant writings and research publications that have originated over many centuries and that illustrate how the knowledge on the condition slowly accumulated.” (p. 1) |
| Kromberg JGR. Psychosocial and cultural aspects of albinism. In: Kromberg JGR and Manga P (eds) *Albinism in Africa: Historical, geographic, medical, genetic, and psychosocial aspects*. Academic Press, 2018, pp.171-201. | Book chapter | Human Genetics | Africa |  | “Two specific aspects of albinism underlie the discussions covered in this chapter. The first is the relationship between the affected person and his physique, or the somatopsychological problems, and how factors within the person ameliorate or negatively affect adjustment. The second involves the social factors that encompass the affected individual and how they impact on that individual, either aiding adjustment or contributing to maladjustment. Together these issues, which are inextricably intertwined, are termed psychosocial aspects.” (p. 172) |
| Kromberg JGR. Genetic counseling and albinism. In: Kromberg JGR and Manga P (eds) *Albinism in Africa: Historical, geographic, medical, genetic, and psychosocial aspects.* Academic Press, 2018, pp.203-233. | Book chapter | Human Genetics | South Africa | Africa | “This chapter will cover a broad view of the field of genetic counseling because it is little known in Africa, its benefits are not yet recognized, and literature on the topic is very scarce.” (p. 206) |
| Kromberg JGR and Kerr R. Genetic testing, postnatal, and prenatal diagnosis for albinism. In: Kromberg JGR and Manga P (eds) *Albinism in Africa: Historical, geographic, medical, genetic, and psychosocial aspects.* Academic Press, 2018, pp.235-256. | Book chapter | Human Genetics | South Africa | Africa | “Such testing should be accompanied by genetic counseling because full information should be provided and informed decisions should be made. When an affected fetus is identified, some couples might continue the pregnancy and others might request termination (legal in such situations in South Africa). Ethical issues that arise in the offering of genetic services and prenatal testing include respect for autonomy, equal access to high-quality services, the offering of options, and freedom of choice.” (p. 235) |
| Kromberg JGR. Interventions: Preventive management, empowerment, advocacy, and support services. In: Kromberg JGR and Manga P (eds) *Albinism in Africa: Historical, geographic, medical, genetic, and psychosocial aspects*. Academic Press, 2018, pp.271-293. | Book chapter | Human Genetics | South Africa | Africa | “This chapter will highlight issues associated with the management of albinism.” [Preventive management, Empowerment, Advocacy, and Support Services) (p. 272) |
| Likumbo N. *Experiences perceptions and understanding of mothers of children living with albinism in Malawi: A qualitative descriptive study*. Master's thesis, University of Cape Town, 2019. | Thesis | Nursing | South Africa | Malawi | "To explore and describe the experiences, perceptions and understanding of mothers who have children living with albinism in Malawi." (p. vi) |
| Lund, P. (2017). *What we can do about the impact of stigma on the lives of people with albinism? Recommendations*. https://www.firah.org/upload/l-appel-a-projets/projets-laureats/2018/albinisme/recommendations-albinism.pdf | authors | human genetics, NGOs | UK | Tanzania and Uganda | This report provides recommendations based on their project to various government stakeholders including Ministry of Health and Ministry of Education. |
| Mhando NE. *Evaluation of the impact of the UNESCO intervention relating to people with albinism carried out within the framework of the UN Development Assistance Plan* (UNDAP 2011-2016) in Tanzania, https://en.unesco.org/sites/default/files/impact_evaluation_-_people_with_albinism.pdf (2016, accessed December 16 2024). | International organization | n/a | From an international organization but through the UNESCO Dar es Salaam office | Tanzania | "The overall objective of the project is to utilise community based strategies to promote the protection of people with albinism and the prevention of further stigma, discrimination and violence affecting their daily lives. It aims to implement institutional change through educating and mobilising local Government to act to promote and protect the rights of people with albinism and to work through media interventions and with key community stakeholders in order to change mind-sets regarding people with albinism. Specifically, the project objectives are: i. Empower local Government and local leaders to mobilise and sensitise communities in relation to people with albinism through utilisation of the socio-cultural approach. ii. To promote positive attitudes, perceptions and practices towards people with albinism amongst community and family members iii. To improve quality of services and care provided to children with albinism at Buhangija and Mitindo primary schools through training of care givers" (p. 9) |
| Ngula A. *The information needs of people with albinism in Khomas Region, Namibia*. Master's thesis, University of Cape Town, 2018. | Thesis | Philosophy (librarian) | Namibia | Namibia | "The main objective of this study was to investigate the information needs of PWA in Khomas region, Namibia, in order to inform the design of their information services… the study specifically sought to:  · Ascertain the information needs of PWA, including the kind of information needs they have;  · Establish which mechanisms PWA use to seek information that meets their information needs; and who initiates their information activity;  · Identify the challenges PWA in Khomas region face daily when seeking information; and  · Determine information services that are appropriate for PWA in Khomas region." (p. 12-13) |
| Pooe-Monyemore MBJ. *A model for enhancement of self-concept of people with albinism*. PhD thesis, University of South Africa, 2007. | Authors | Literature and Philosophy, health studies | South Africa | South Africa and Zimbabwe | “The researcher wished to answer the following research questions:  • What is the life experience of people with oculocutaneous albinism and what does it mean to them to have the condition?  • What could be done in the clinical practice to improve the quality of life of people with oculocutaneous albinism?” (p. 4) |
| Reimer-Kirkham S, Mooa RS, Astle B, et al. *Mothering and albinism: Recommendations to support mothers and children impacted by albinism in South Africa,* https://motheringandalbinism.com/wp-content/uploads/2024/02/mothering-and-albinism-report-digital-final1.pdf (2023, accessed December 17 2024). | authors | Nursing, advocates, human rights | Canada & South Africa | South Africa | "This report provides recommendations to support persons with albinism and their families, focusing on social responses and advocacy, as well as government policies and resources to improve the livelihood of mothers and their children affected by albinism." (p. 2) |
| Source of the Nile Union of Persons with Albinism, Advantage Africa, Coventry University, et al. *"We are humans too!": What we can do about the impact of stigma on the lives of people with albinism,* https://africaalbinismnetwork.org/wp-content/uploads/2022/09/1532564200045dl8al7mta43h37tpyulp36jemi-1.pdf (n.d., accessed December 16 2024). | NGOs | n/a | UK, Uganda | Uiganda | This report is based off of a research conducted in Uganda and It provides recommendations for stakeholders. |
| Strobell E. *Exploring the experience of mothers who have children with albinism in Tanzania: A critical* *ethnography*. Trinity Western University, 2020. | Thesis | nursing | Canada | Tanzania | "A focused critical ethnographic study, through the lens of Hudson-Weems’ (2019) Africana Womanism, explored the experiences of mothers of children with albinism in Tanzania, addressed the gendered nature of this condition, and considered the human rights and resilience of the mothers" (p. 7). |
| Tanzania Albinism Society and Karagwe Community Based Rehabilitation Programmes. *The 2019 baseline survey on socio-economic status of persons with albinism and their households in the Lake Zone,* https://uprdoc.ohchr.org/uprweb/downloadfile.aspx?filename=8754&file=EnglishTranslation (2019, accessed December 16 2024). | NGO and govt | n/a | Tanzania | Tanzania | "The 2019 Baseline Survey aimed at examining the socio-economic status of persons with albinism and their households in the Lake Zone. It aimed specifically to determine living conditions and to evaluate statistical information concerning healthcare services, education, employment, participation and security of PwA and their households." (p. xix) |
| Thuku M. *Myths, discrimination, and the call for special rights for persons with albinism in Sub-Saharan Africa*, http://www.underthesamesun.com/sites/default/files/MYTHS.Final_.pdf (2021, accessed December 16 2024). | Author | unknown | unknown | sub-Saharan Africa | "This paper looks at the myths surrounding albinism and the resulting discrimination. In response to these vices, it proposes some special rights that states in Africa should implement with regards to health, education, livelihood, safety and security of the person and the general welfare of persons with albinism. References have been made to various papers and articles published online. The paper is intended to be a resource for persons/organizations defending the rights of persons with albinism in sub-Saharan Africa." (p. 1) |
| Tinkham C. *Albinism in Africa: Examining identity formulation*. Master's thesis, University of Tennessee at Chattanooga, 2021. | Thesis | Anthropology | USA | Africa | “This paper, based on an extensive literature review, explores and discusses the social and cultural significances of albinism in Sub-Saharan Africa, as well as the identity-building process that people with albinism (PWA) encounter, a battle beginning with infancy that remains a source of adversity for all of one’s life, evident through unequal access to medical treatment, school and work place discrimination, social isolation, and subjugation to the negative consequences of superstitious beliefs.” (p. 4) |
| Tjope M. *Life with albinism filled with pearls*. Mpho Tjope, 2015. | Book | albinism advocate | South Africa | South Africa/ Africa | This book shares various experiences of PWA in Africa, including the challenges they faced. |
| Udongo BP, Bagonza. A. and Namutebi O. *Spatial mapping and profiling of persons with albinism in Eastern Uganda: Report on pilot study of persons with albinism in the districts of Budaka, Bududa, Butaleja, Buyende, Kamuli, Kumi, Manafwa, Mayuge, Sironko, and Soroti.* Albinism Umbrella, https://voice.global/assets/2019/06/Abridged-version-of-Spartial-mapping-Report-.pdf (2018, accessed December 16 2024). | NGO | n/a | Uganda | Uganda | "The main aim of the study is to create a data base through mapping all Persons with Albinism and organisations serving these people in 10 selected districts of Eastern Uganda." (p. 2) |
| Under the Same Sun. *Children with albinism in Africa: Murder mutilation and violence,* https://underthesamesun.com/sites/default/files/UTSS%20report%20to%20UN%20-%20REPORT.pdf (2012, accessed December 16 2024). | International organization | n/a | International but has a base in Tanzania | Tanzania | "The intent of the report is to present a detailed and comprehensive overview of the situation in Tanzania concerning persons living with albinism (PWA), particularly children. It is hoped that the report will assist Ms. Santos Pais in her mandate and reports on violence against children." (p. 7) |
| Under the Same Sun. *History of attacks against persons with Albinism (PWA),* https://www.underthesamesun.com/wp-content/uploads/2022/08/History-of-Attacks-against-PWA.pdf (2013, accessed December 16 2024). | International organization | n/a | International but has a base in Tanzania | "Particular focus on the region of Sub-Saharan Africa" (p. 1) | "This paper takes on an enormous task. It attempts to explain the history and origins of the attacks against persons with albinism (PWA). In light of the monstrosity of this subject matter, this paper can only scratch the surface of the issue." (p. 1) |
| Under the Same Sun. *Human rights violations on children with albinism in Africa 2015-2016,* https://africaalbinismnetwork.org/wp-content/uploads/2022/09/1604597087348lxxtyfws97j-1.pdf (2016, accessed December 16 2024). | International organization | n/a | International but has a base in Tanzania | Africa | The report describes some of the cases of attacks against PWA as well as other challenges they faced. "Under the Same Sun makes recommendations in the areas of education, healthcare, protection, legal assistance, policy, awareness raising, and cooperation with international human rights mechanisms to address violation of human rights of children with albinism in Africa." (p. 1) |
| Under the Same Sun. *Reported attacks re. persons with albinism,* https://www.underthesamesun.com/wp-content/uploads/2023/07/Attacks-of-PWA-Extended-07.05.23.pdf (2023, accessed December 16 2024). | International organization | n/a | International but has a base in Tanzania | Worldwide but primarily in Africa | "UTSS gathers this data from its own field research and also from its partners on the ground. We record cases that have been thoroughly verified" (p. 1) |
| United Nations Educational Scientific and Cultural Organization. *Report on the public hearings on discrimination and other challenges faced by persons with albinism in Namibia,* https://namibia.un.org/en/202926-report-public-hearings-discrimination-and-other-challenges-faced-persons-albinism-namibia (2022, accessed December 16 2024). | International organization | n/a | n/a | Namibia | "This report outlines how the public hearings were conducted, their purpose, and their outcomes. It further investigates discrimination against people with albinism, customary practices and myths, violence and other challenges. Recommendations and possible solutions are also included." (p. 2) |
| United Nations General Assembly. *Persons with albinism: Report of the Office of the United Nations High*  *Commissioner for Human Rights* (A/HRC/24/57), https://undocs.org/Home/Mobile?FinalSymbol=A%2FHRC%2F24%2F57&Language=E&DeviceType=Desktop&LangRequested=False (2013, accessed July 7 2024). | International organization | n/a | n/a | Worldwide but focused on Africa | “The present report provides an overview of the most serious human rights violations faced by persons with albinism, primarily focusing on the ritual killings and attacks to which they are subjected. It also highlights the multiple forms of discrimination, stigma and social exclusion faced by persons with albinism which occur across the globe.” (p. 3) |
| United Nations General Assembly. *Preliminary survey on the root causes of attacks and discrimination against persons with albinism* (A/71/255), https://documents-dds-ny.un.org/doc/UNDOC/GEN/N16/241/46/PDF/N1624146.pdf?OpenElement (2016, accessed July 7 2024). | International organization | n/a | n/a | Worldwide | "In the present report, the Independent Expert considers how interrelated factors, including myths, poverty, witchcraft practices and other aggravating factors such as visibility and appearance, contribute to ongoing outbreaks of attacks against persons with albinism and exacerbate pre existing contexts of discrimination and stigma." (p. 1) |
| United Nations General Assembly. *Report of the Independent Expert on the enjoyment of human rights by persons with albinism on her mission to Malawi* (A/HRC/34/59/Add.1), https://documents-dds-ny.un.org/doc/UNDOC/GEN/G16/433/28/PDF/G1643328.pdf?OpenElement (2016, accessed July 7 2024). | International organization | n/a | n/a | Malawi | "In her report, the Independent Expert on the enjoyment of human rights by persons with albinism explores issues relating to the rights of persons with albinism in Malawi. On the basis of information gathered prior to and during her visit, the Independent Expert focuses on subsisting challenges in the context of the recent wave of attacks against persons with albinism in the country. The Independent Expert also discusses other aspects of the rights of persons with albinism in Malawi such as discrimination, including in the area of health rights, as well as access to education. She highlights the important steps taken by the Government to address some of these issues and makes recommendations to strengthen efforts, address implementation gaps and remove barriers to the enjoyment of human rights by Malawians with albinism." (p. 1) |
| United Nations General Assembly. *Vision of the mandate* (A/HRC/31/63), https://undocs.org/Home/Mobile?FinalSymbol=A%2FHRC%2F31%2F63&Language=E&DeviceType=Desktop&LangRequested=False (2016, accessed July 7 2024). | International organization | n/a | n/a | Worldwide | "In the report, the Independent Expert presents a number of reflections on the situation of the enjoyment of human rights by persons with albinism as well as on her vision for her work as Independent Expert, including how she intends to fulfill the requirements of her mandate and the issues she wishes to address as a matter of priority." (p. 1) |
| United Nations General Assembly. *Applicable international human rights standards and related obligations addressing the issues faced by persons with albinism* (A/72/131), https://undocs.org/Home/Mobile?FinalSymbol=A%2F72%2F131&Language=E&DeviceType=Desktop&LangRequested=False (2017, accessed July 7 2024). | International organization | n/a | n/a | Worldwide | "In the present report, the Independent Expert on the enjoyment of human rights by persons with albinism looks at the international human rights standards pertaining to the human rights violations faced by persons with albinism and the correlating obligations for States." (p. 1) |
| United Nations General Assembly. *Witchcraft and the human rights of persons with albinism* (A/HRC/34/59), https://documents-dds-ny.un.org/doc/UNDOC/GEN/G17/004/01/PDF/G1700401.pdf?OpenElement (2017, accessed July 7 2024). | International organization | n/a | n/a | Worldwide | "In her report the Independent Expert provides a brief account of activities undertaken in fulfilment of the mandate in 2016. She also discusses witchcraft as a root cause of attacks against persons with albinism, the impact of witchcraft on the enjoyment of human rights by persons with albinism and ways forward." (p. 1) |
| United Nations General Assembly. *Right of persons with albinism to the highest attainable standard of health* (A/HRC/37/57), https://documents-dds-ny.un.org/doc/UNDOC/GEN/G17/364/80/PDF/G1736480.pdf?OpenElement (2018, accessed July 7 2024). | International organization | n/a | n/a | Worldwide | "In her report, the Independent Expert explores matters concerning the enjoyment of the right to the highest attainable standard of health by persons with albinism. On the basis of information gathered from surveys, reports and country visits, she conceptualizes the multifaceted challenges in the field of health, including disabilities arising from visual impairment and the high number of fatalities caused by skin cancer in certain regions, the applicable norms and standards in international human rights law, the continuing challenges and best practices." (p. 1) |
| United Nations General Assembly. A/HRC/43/42: *Women and children impacted by albinism - Report of the Independent Expert on the enjoyment of human rights by persons with albinism,* https://www.ohchr.org/en/documents/thematic-reports/ahrc4342-women-and-children-impacted-albinism-report-independent-expert (2019, accessed December 4 2024). | International organization | n/a | n/a | Worldwide but focused on Africa | "In the present report, the Independent Expert on the enjoyment of human rights by persons with albinism provides an overview of the situation of women and children impacted by albinism in different regions of the world. She identifies barriers to the enjoyment of human rights in this context, as well as best practices and recommendations." (p. 1) |
| United Nations General Assembly. *Study on the situation of the violations and abuses of human rights rooted in harmful practices related to accusations of witchcraft and ritual attacks, as well as stigmatization* (A/HRC/52/47), https://undocs.org/Home/Mobile?FinalSymbol=A%2FHRC%2F49%2F56&Language=E&DeviceType=Desktop&LangRequested=False (2023, accessed July 7 2024). | International organization | n/a | n/a | Worldwide | "In it the Office of the United Nations High Commissioner for Human Rights depicts the severity of human rights violations and abuses rooted in harmful practices related to accusations of witchcraft and ritual attacks. It also describes the adverse human rights impacts on persons in vulnerable situations and the factors that affect their vulnerability" (p. 1) |
