# Supplementary material for: A participatory systematic review on human rights and the birth of a child with albinism in sub-Saharan Africa
Source: Womens Health (Lond). 2025 Dec 11;21:17455057251395420. doi: 10.1177/17455057251395420 (PMC12699010; doi:10.1177/17455057251395420)
Supplement: sj-docx-3-whe-10.1177_17455057251395420 – Supplemental material for A participatory systematic review on human rights and the birth of a child with albinism in sub-Saharan Africa [file sj-docx-3-whe-10.1177_17455057251395420.docx]

**Supplemental File: Quality Appraisals**

**Quality Appraisal of Qualitative Research Articles**

(Level of Evidence for Meaningfulness = 1 as qualitative research)

| **Study** | **Q1** | **Q2** | **Q3** | **Q4** | **Q5** | **Q6** | **Q7** | **Q8** | **Q9** | **Q10** | **Total Yes Responses** |
| --- | --- | --- | --- | --- | --- | --- | --- | --- | --- | --- | --- |
| Baker et al. (2010) | Y | Y | Y | Y | Y | Y | Y | Y | U | Y | 9 |
| Braathen & Instead (2006) | Y | Y | Y | Y | Y | N | N | Y | U | Y | 7 |
| Bradbury-Jones et al. (2018) | Y | Y | Y | Y | Y | Y | Y | Y | Y | Y | 10 |
| Brocco (2015) | Y | Y | Y | Y | Y | N | Y | Y | U | Y | 8 |
| Brocco (2016) | Y | Y | Y | Y | Y | N | N | Y | Y | Y | 8 |
| Ibhawoh et al. (2022) | Y | Y | Y | Y | Y | Y | Y | Y | Y | Y | 10 |
| Kayombo (2021) | Y | Y | Y | Y | Y | N | Y | Y | Y | Y | 9 |
| Kiluwa et al. (2022) | Y | Y | Y | Y | Y | U | N | Y | Y | Y | 8 |
| Kromberg et al. (1987) | Y | Y | Y | Y | Y | Y | Y | Y | Y | Y | 10 |
| Likumbo et al (2021) | Y | Y | Y | Y | Y | Y | U | Y | Y | Y | 9 |
| Lynch et al. (2014) | Y | Y | Y | Y | Y | N | U | Y | U | Y | 7 |
| Machoko (2013) | Y | Y | Y | Y | Y | N | N | Y | U | Y | 7 |
| Ngula (2023) | Y | Y | Y | Y | Y | Y | Y | Y | U | Y | 9 |
| Nyamu (2020) | Y | Y | Y | Y | Y | Y | Y | Y | Y | Y | 10 |
| Reimer-Kirkham et al. (2020) | Y | Y | Y | Y | Y | Y | Y | Y | Y | Y | 10 |
| Reimer-Kirkham et al. (2024) | Y | Y | Y | Y | Y | Y | Y | Y | Y | Y | 10 |
| Tambala-Kaliati et al. (2021) | Y | Y | Y | Y | Y | N | N | Y | Y | Y | 8 |
| Taylor et al. (2021) | Y | Y | Y | Y | Y | Y | Y | Y | Y | Y | 10 |
| Number of studies receiving a Yes (%) | 18  (100%) | 18  (100%) | 18  (100%) | 18  (100%) | 18  (100%) | 10  (55.5%) | 11  (61%) | 18  (100%) | 12  (66.7%) | 18  (100%) |  |

Y= Yes; N= No; U= Unclear; N/A= Not applicable

**JBI Critical Appraisal Checklist for Qualitative Research:**

Q1 . Is there congruity between the stated philosophical perspective and the research methodology?

Q2 . Is there congruity between the research methodology and the research question or objectives?

Q3   Is there congruity between the research methodology and the methods used to collect data?

Q4 . Is there congruity between the research methodology and the representation and analysis of data?

Q5 . Is there congruity between the research methodology and the interpretation of results?

Q6 . Is there a statement locating the researcher culturally or theoretically?

Q7 . Is the influence of the researcher on the research, and vice- versa, addressed?

Q8 . Are participants, and their voices, adequately represented?

Q9 . Is the research ethical according to current criteria or, for recent studies, is there evidence of ethical approval by an appropriate body?

Q10 . Do the conclusions drawn in the research report flow from the analysis, or interpretation, of the data?

**Quality Appraisal of Discussion Articles**

(Level of Evidence for Meaningfulness = 5 as expert opinion)

| **Study** | **Q1** | **Q2** | **Q3** | **Q4** | **Q5** | **Q6** | **Total Yes Responses** |
| --- | --- | --- | --- | --- | --- | --- | --- |
| Aquaron et al. (2009) | Y | Y | Y | Y | Y | Y | 6 |
| Benyah (2017) | Y | Y | Y | Y | Y | Y | 6 |
| Brocco (2021) | Y | Y | Y | Y | Y | Y | 6 |
| Bryceson et al. (2010) | Y | Y | Y | U | Y | Y | 5 |
| Cruz-Inigo et al. (2011) | Y | Y | Y | Y | Y | Y | 6 |
| De Pina-Cabral (2013) | Y | Y | Y | Y | Y | Y | 6 |
| Imafidon (2017) | Y | Y | Y | Y | Y | Y | 6 |
| Kromberg & Kerr (2022) | Y | Y | Y | Y | Y | Y | 6 |
| Kromberg et al. (2020) | Y | Y | Y | Y | Y | Y | 6 |
| Lund (2005) | Y | Y | Y | Y | Y | Y | 6 |
| Mostert (2019) | Y | Y | Y | Y | Y | Y | 6 |
| Munyere (2004) | Y | Y | Y | Y | N | N | 4 |
| Ojilere & Saleh (2019) | Y | Y | Y | Y | Y | Y | 6 |
| Reimer-Kirkham et al. (2022) | Y | Y | Y | Y | Y | Y | 6 |
| Reimer-Kirkham et al. (2021) | Y | Y | Y | Y | Y | Y | 6 |
| Taylor et al. (2019) | Y | Y | Y | Y | Y | Y | 6 |
| Number of studies receiving a Yes (%) | 16 | 16 | 16 | 15  (93.8%) | 16 | 15  (93.8%) |  |

Y= Yes; N= No; U= Unclear; N/A= Not applicable

**JBI Critical Appraisal Checklist for Text and Opinion Papers**

Q1 . Is the source of the opinion clearly identified?

Q2 . Does the source of opinion have standing in the field of expertise?

Q3   Are the interests of the relevant population the central focus of the opinion?

Q4 . Is the stated position the result of an analytical process, and is there logic in the opinion expressed?

Q5 . Is there reference to the extant literature?

Q6 . Is any incongruence with the literature/sources logically defended?

**Quality Appraisal of Systematic Review Articles**

(Level of Evidence for Meaningfulness = 2 as mixed-methods synthesis)

| **Study** | **Q1** | **Q2** | **Q3** | **Q4** | **Q5** | **Q6** | **Q7** | **Q8** | **Q9** | **Q10** | **Q11** | **Total Yes Responses** |
| --- | --- | --- | --- | --- | --- | --- | --- | --- | --- | --- | --- | --- |
| Reimer-Kirkham et al. (2019) | Y | Y | Y | Y | Y | U | Y | Y | U | Y | Y | 9 |

Y= Yes; N= No; U= Unclear; N/A= Not applicable

**JBI Critical Appraisal Checklist for Systematic Review Papers**

Q1 . Is the review question clearly and explicitly stated?

Q2 . Were the inclusion criteria appropriate for the review question?

Q3   Was the search strategy appropriate?

Q4 . Were the sources and resources used to search for studies adequate?

Q5 . Were the criteria for appraising studies appropriate?

Q6 . Was critical appraisal conducted by two or more reviewers independently?

Q7. Were there methods to minimize errors in data extraction?

Q8. Were the methods used to combine studies appropriate?

Q9. Was the likelihood of publication bias assessed?

Q10. Were recommendations for policy and/or practice supported by the reported data?

Q11. Were the specific directives for new research appropriate?
