# Supplementary material for: A participatory systematic review on human rights and the birth of a child with albinism in sub-Saharan Africa
Source: Womens Health (Lond). 2025 Dec 11;21:17455057251395420. doi: 10.1177/17455057251395420 (PMC12699010; doi:10.1177/17455057251395420)
Supplement: sj-docx-4-whe-10.1177_17455057251395420 – Supplemental material for A participatory systematic review on human rights and the birth of a child with albinism in sub-Saharan Africa [file sj-docx-4-whe-10.1177_17455057251395420.docx]

**Supplemental File: Codebook**

| **Name** | **Description** |
| --- | --- |
| A. Birth experience | Birth experience, including clinical nature of birth (natural c-section, complications) |
| B. Family history of albinism |  |
| C. Reactions to a baby with albinism | [Do not code here] |
| C1. Reactions of mother | This code is for when the experience is shared by a participant. This includes initial reactions and gradual acceptance (over time). Can be shared (as participants) by a mother, family, birth attendants, NGO/CSO personnel |
| C1a. Insights relating to maternal-infant bonding |  |
| C1b. Mental health of mother | i.e., postnatal depression, anxiety |
| C2. Reactions of father | This code is for when the experience is shared by a participant. This includes neglecting the child and abandonment. |
| C3. Reactions of family | This code is for when the experience is shared by a participant. I.e., excluded, abandoned |
| C4. Reactions of birth attendants (nurses, midwives, physicians, birth attendants) | This code is for when the experience is shared by a participant. Can be shared (as participants) by a mother, family, birth attendants, NGO/CSO personnel |
| C5. Reactions of Community | This code is for when the experience is shared by a participant. This includes experiences of mothers and babies experiencing stigma, discrimination, exclusion. Can be shared (as participants) by a mother, family, birth attendants, NGO/CSO personnel |
| C6. General broad reactions (discussions) | This code is for generalized discussions about the reactions of families and communities Iin contrast to the above that are insights from particular people / participants). May be from the background of the paper |
| D. Settings of birth |  |
| D1. Birthing setting | Place of birth, i.e., hospital, clinic, home Context of place, i.e., urban/rural Any context regarding birth |
| D2. Birthing traditions | Any traditions that involves a birth, i.e., ceremonies, cultural activities |
| E. Resources | [Do not code here] |
| E1. Provided with education prior to discharge | Mother or family provided with education prior to discharge |
| E2. Referrals | Mother or family provided with referrals to genetic counselling, ophthalmologist, dermatologist, social worker, mental health counselling |
| E3. Connected to an NGO support group | Mother or family are connected to an NGO support group. |
| F. Explanation about albinism | [Do not code here] |
| F1. Genetic understanding of albinism | (or the lack of) |
| F2. Explanation about the birth of a baby with albinism (non-scientific) | This includes accusations of mother’s infidelity, mother brought a curse to family/community |
| F3. Beliefs about PWA | Generally referring to beliefs about PWA, not necessarily about their birth |
| G. Contextual Factors | [Do not code here] |
| G1. Supportive Factors that helped mothers and families | (basket code) |
| G2. Unsupportive Factors that harmed mothers and families | (basket code) |
| H. Perspectives | [Do not code here] |
| H1. Gender |  |
| H2. Human Rights |  |
| H3. Colourism |  |
| H4. Disability |  |
| H5. Religion | Reference to African traditional religions, faith communities |
| H6. Other |  |
| I. Violence | [Do not code here] |
| I1. Gender-based violence | Towards the mother |
| I2. Infant violence | Towards the baby, i.e., infanticide |
| J. Insights to changes shifts over time | “in contrast to earlier study”, “previous studies described”, or with an earlier albinism birth |
| K. Health professions education | [Do not code here] |
| K1. Insights about the nature (content, approach) of health professions education | Knowledge of birth attendants (or lack of) about albinism Providing or improving education of nurses, midwives, doctors, etc. |
| K2. Reference to Respectful Maternity Care (or its concepts) | Micro level: point of care, power and control, abuse  Meso level: are midwives equipped, what is their work environment  Macro level: govt/ policy |
| L. Recommendations | [Do not code here] |
| L1. Implications for healthcare providers | e.g., providing education to families, have positive responses |
| L2. Policy Improvement |  |
| L3. Collaboration among stakeholders |  |
| L4. Awareness Raising |  |
| L5. Other |  |
