# Supplementary material for: A participatory systematic review on human rights and the birth of a child with albinism in sub-Saharan Africa
Source: Womens Health (Lond). 2025 Dec 11;21:17455057251395420. doi: 10.1177/17455057251395420 (PMC12699010; doi:10.1177/17455057251395420)
Supplement: sj-docx-5-whe-10.1177_17455057251395420 – Supplemental material for A participatory systematic review on human rights and the birth of a child with albinism in sub-Saharan Africa [file sj-docx-5-whe-10.1177_17455057251395420.docx]

**Supplemental File: Data Extraction Form for Academic and Grey Literature**

**Academic Literature**

| **Birthing Stories: Participatory Systematic Review** | | |
| --- | --- | --- |
| Reviewer | Date | |
| **Publication Information** | | |
| Title | | |
| Country Focus | | Year |
| **Setting of birth, i.e., hospital, clinic, at home (if applicable)** | | |
|  | | |
| **Participants’ Characteristics (i.e., stakeholders, mothers, healthcare providers)** | | |
|  | | |
| **Who was in the room during the birth (i.e., partner, nurses, midwives)?** | | |
|  | | |
| **What was the initial reaction of the mother?** | | |
|  | | |
| **What were the initial reactions of the partner and family members?** | | |
|  | | |
| **What were the initial reactions of the healthcare providers)?** | | |
|  | | |
| **Explanations of the birth of a baby with albinism** | | |
|  | | |
| 1. **Was the family provided education about albinism prior to leaving the hospital?** 2. **Were they referred to other healthcare providers (i.e., genetic counselling, social worker, mental health counselling, dermatologist, ophthalmologist)?** 3. **Were mothers and their families connected to an NGO or support group (which group)?**   **If yes, please explain.** | | |
|  | | |
| **What were the supportive factors that helped the mothers and their families?** | | |
|  | | |
| **What were the unsupportive factors from families and healthcare providers?** | | |
|  | | |
| **Were there specific insights relating to gender? If yes, please explain.** | | |
|  | | |
| **Were there insights relating to the bonding of the mother and baby? If yes, please explain.** | | |
|  | | |
| **Were there insights related to historical or contextual changes that influenced the mothers, families, and healthcare providers’ experiences with a birth to a baby with albinism? If yes, please explain.** | | |
|  | | |
| **Did the article provide insights into health professions education? If yes, please explain.** | | |
|  | | |
| **What was the conceptual framework? Are these concepts drawn upon (i.e., human rights, disability, colourism, respectful maternity care)?** | | |
|  | | |
| **Implications – What recommendations did the author(s) have?** | | |
|  | | |
| **Future research (if applicable)** | | |
|  | | |

**Grey Literature**

| **Birthing Stories: Participatory Systematic Review** | | |
| --- | --- | --- |
| Reviewer | Date | |
| **Publication Information** | | |
| Title | | |
| Country Focus | | Year |
| Organization or government name | | |
| **Setting of birth, i.e., hospital, clinic, at home (if applicable)** | | |
|  | | |
| **Participants’ Characteristics (i.e., stakeholders, mothers, healthcare providers, if applicable)** | | |
|  | | |
| **Who was in the room during the birth (i.e., partner, nurses, midwives)?** | | |
|  | | |
| **What was the initial reaction of the mother?** | | |
|  | | |
| **What were the initial reactions of the partner and family members?** | | |
|  | | |
| **What were the initial reactions of the healthcare providers)?** | | |
|  | | |
| **Explanations to the birth of a baby with albinism** | | |
|  | | |
| 1. **Was the family provided education about albinism prior to leaving the hospital?** 2. **Were they referred to other healthcare providers (i.e., genetic counselling, social worker, mental health counselling, dermatologist, ophthalmologist)?** 3. **Were they connected to an NGO or support group (which group)?**   **If yes, please explain.** | | |
|  | | |
| **What were the supportive factors that helped the mothers and their families?** | | |
|  | | |
| **What were the unsupportive factors from families and healthcare providers?** | | |
|  | | |
| **Were there specific insights relating to gender? If yes, please explain.** | | |
|  | | |
| **Were there insights relating to the bonding of the mother and baby? If yes, please explain.** | | |
|  | | |
| **Were there insights related to historical or contextual changes that influenced the mothers, families, and healthcare providers’ experiences with a birth to a baby with albinism? If yes, please explain.** | | |
|  | | |
| **Did the article provide insights to health professions education?** | | |
|  | | |
| **Are concepts such as human rights, disability, colourism, and respectful maternity care drawn upon?** | | |
|  | | |
| **Implications – What recommendations did the author(s) have for government, CSOs, healthcare providers?** | | |
|  | | |
| **Future research** | | |
|  | | |
