# Supplementary material for: A participatory systematic review on human rights and the birth of a child with albinism in sub-Saharan Africa
Source: Womens Health (Lond). 2025 Dec 11;21:17455057251395420. doi: 10.1177/17455057251395420 (PMC12699010; doi:10.1177/17455057251395420)
Supplement: sj-docx-6-whe-10.1177_17455057251395420 – Supplemental material for A participatory systematic review on human rights and the birth of a child with albinism in sub-Saharan Africa [file sj-docx-6-whe-10.1177_17455057251395420.docx]

**Supplemental File: Health Education on Albinism**

**WHAT IS ALBINISM?**

Albinism is a hereditary, genetic condition that results in the lack of pigmentation (also known as melanin) in the hair, skin and eyes. There is no cure for albinism.

**WHAT CAUSES ALBINISM?**

Albinism is caused by a **recessive gene**, meaning **both parents need to carry the gene** for a child to be born with albinism. The parents do not need to have albinism themselves. Siblings without albinism may be carriers of the gene. Albinism is not caused by a curse or wrongdoing by the mother or family.

**HOW COMMON IS ALBINISM?**

Albinism occurs worldwide, regardless of gender, racial or ethnic backgrounds.

Frequency varies worldwide. Here are some estimates:

Cameroon: 1 in 20, 000 have albinism

Namibia: 1 in 1,755

South Africa: 1 in 3,900

Swaziland: 1 in 1900

Tanzania: 1 in 2673

Zimbabwe: 1 in 4728

Denmark: 1 in 14000

Netherlands: 1 in 15000

United States of America: 1 in 17000

**CONSEQUENCES OF ALBINSIM**

People with albinism often appear pale compared to their family members. They often have blonde or sandy hair, white skin and light eyes.

Albinism leads to low vision or vision impairment. It also results in sensitiveity to the sun and bright light. Persons with albinism are also vulnerable to sun burn and skin cancer if they do not use sunscreen or were sun protective clothing, carry umbrellas for protection. Stigma from others often leads to discrimination against people with albinism. It is important that they are treated with respect and dignity as they are human like every other person. With support, persons with albinism excel like everyone else

**HEALTH SUPPORT FOR PERSONS WITH ALBINISM?**

**Information**

- Provide parents with this information about albinism to combat stigma and foster acceptance and love of their child with albinism
- Inform key persons and groups within the child’s community such as her village chiefs, community leaders, religious leaders, educators, health workers, neighbours and so on.

**Eye**

Refer to an eye doctor

- People with albinism often will need **eyeglasses** as well as sunglasses for UV (ultraviolet) protection.
- Even with eyeglasses, they may still experience impaired vision.
- It is recommended they see an eye doctor before they start school.

**Skin**

Refer to a skin doctor or dermatologist. **Avoid the sun**.

- Avoid the sun
- If avaible, use sunscreen lotion with at least 50 SPF (sun protection factor) on all areas exposed such as face, neck and hands.
- Wear clothing and hat that completely cover the skin. Use dark umbrellas if possible.
- Visit the doctor or dermatologist for regular screening **for sun-related skin damage and skin cancer** (e.g., family doctor, dermatologist).

**Other Support**

- Genetic Counselling: Parents require education support (genetic counselling) about the cause of albinism and the needs of a child with albinism.
- Counselling: Consider referring the parents to a mental health counsellor for emotional support and social worker for social and health access, including applicable grants that are available.
- Meeting Others: Consider connecting families with other persons with albinism or families who live with albinism (e.g., non-governmental organizations, support groups).
